# Investigating the association between interferons and flares of systemic lupus erythematosus at a single cell level

**DOI:** 10.3389/fimmu.2026.1901515

**Published:** 2026-10-06

**Authors:** Zoha Faheem, Giselle M. Boukhaled, Carol Nassar, Kieran Manion, Carolina Munoz-Grajales, Michael Kim, Dennisse Bonilla, Dafna D. Gladman, Laura Whitall-Garcia, Zahi Touma, David G. Brooks, Joan Wither

**Affiliations:** 1 Schroeder Arthritis Institute, University Health Network, Toronto, ON, Canada; 2 Princess Margaret Cancer Centre, University Health Network, Toronto, ON, Canada; 3 Toronto General Hospital Research Institute, University Health Network, Toronto, ON, Canada; 4 Departments of Internal Medicine and Immunology, Rady Faculty of Health Sciences, University of Manitoba, and Manitoba Centre for Proteomics and Systems Biology, Winnipeg, MB, Canada; 5 University of Toronto Lupus Clinic, Centre for Prognosis Studies in Rheumatic Diseases, Division of Rheumatology, Schroeder Arthritis Institute, University Health Network, Toronto, ON, Canada; 6 Department of Medicine, Faculty of Medicine, University of Toronto, Toronto, ON, Canada; 7 Division of Rheumatology, Schroeder Arthritis Institute, University Health Network, Toronto, ON, Canada; 8 Department of Immunology, Faculty of Medicine, University of Toronto, Toronto, ON, Canada

**Keywords:** cellular trafficking, disease flare, interferon, interferon-stimulated proteins, systemic lupus erythematosus

## Abstract

**Background:**

Elevated levels of interferons (IFN) are a hallmark of Systemic Lupus Erythematosus (SLE) and are linked to disease flares, but the precise mechanisms underlying this remain unclear. We examined the association between IFN-stimulated proteins (ISPs) and immune changes in flaring and quiescent SLE patients at single-cell resolution.

**Methods:**

Using CyTOF, six ISPs were measured in PBMC populations from 18 healthy controls, 24 quiescent SLE patients, and 46 recently flaring SLE patients. Individual ISPs and a composite ISP score were assessed.

**Results:**

Analysis of 29 immune populations revealed that mean ISP levels correlated with peripheral blood IFN-stimulated gene (ISG) expression and were higher in flaring than quiescent patients. Despite this correlation, ISP expression was heterogeneous between and within cell subsets. To identify factors contributing to this heterogeneity, we compared the top ISP-expressing cells with the remaining cells in each subject. High ISP-expressing cells had increased trafficking marker expression with distinct expression patterns suggesting that cells may have transited different tissues. Except for β7, these molecules were not induced by IFN *in-vitro*. Longitudinal analysis revealed that ISP expression gradually decreased in T and activated innate immune populations with flare resolution, whereas that in B cell populations remained stable with the baseline levels predicting sustained or recurrent disease activity one year later.

**Conclusion:**

Although mean ISP expression in most cell populations correlated with global ISG expression, there is heterogeneity between and within immune populations in SLE, likely reflecting variable IFN exposure as cells traffic through inflamed sites within the body.

## Introduction

Systemic lupus erythematosus (SLE) is a chronic systemic autoimmune disease with a significant morbidity and mortality. It is highly heterogeneous in the extent and severity of organ involvement, and in most patients follows a relapsing-remitting disease course, with unpredictable periods of exacerbation, known as flares, followed by periods of disease quiescence (1). A hallmark of SLE is the production of autoantibodies, particularly anti-nuclear antibodies (ANAs), which can deposit in tissues and elicit inflammation (2–4). Although mounting evidence indicates that B cells and their progeny, antibody-secreting cells, play a crucial role in disease pathogenesis (5–7), the immunologic mechanisms that drive disease flares have not been fully elucidated.

The majority of SLE patients have elevated levels of interferon (IFN)-stimulated gene (ISG) expression in their peripheral blood (8, 9), which has been associated with disease activity in cross-sectional studies (10–12). Therapeutic administration of Type I IFNs can induce lupus-like disease in a small subset of individuals, and high levels of IFNα or ISG expression in ANA-positive individuals are associated with an increased likelihood of progression to clinical disease (13–15). In longitudinal studies, high baseline levels of ISG expression or IFNα are associated with an increased likelihood of subsequent flare and more severe disease over the following 5 years, as reflected by increased requirements for corticosteroids and immunosuppressive therapies (16, 17). Conversely, treatment of SLE patients with Anifrolumab, a Type I IFN receptor-blocking antibody, is associated with an increased likelihood of attaining a low disease activity state (18).

Although the above findings suggest that IFNs contribute to the development of flares, the precise mechanisms by which they do so remain unclear. IFNs have pleiotropic effects on the immune system, many of which are predicted to exacerbate the immune dysregulation in SLE (19–21). Longitudinal studies by our group and others indicate that the levels of ISG expression in whole peripheral blood remain relatively stable over time, despite changes in disease activity, in the majority of SLE patients (12, 22). However, ISG expression in whole blood cannot discriminate between the different IFN-experienced cell subsets, and it is likely that more abundant populations in the circulation contribute more to global ISG levels than rarer, potentially more pathogenic cell subsets. Furthermore, single-cell RNA sequencing studies on a relatively small number of patients with SLE suggest that distinct circulating immune cell populations have unique ISG signatures, and that the expansion of subpopulations with high ISG signatures may correlate with disease activity (23).

In this study, we used a unique longitudinal cohort of acutely flaring and quiescent patients to investigate the relationship between the whole peripheral blood IFN signature and single cell expression of IFN-stimulated proteins (ISPs), as surrogate markers of ISG expression. By examining ISP expression across immune cell subsets over time, we aimed to gain insight into the cellular mechanisms through which elevated levels of IFNs promote immune dysregulation and disease flares in SLE.

## Materials and methods

### Study participants

Patients ≥18 years old with a diagnosis of SLE, based on the 2019 European League Against Rheumatism/American College of Rheumatology classification criteria (24), were recruited through the University of Toronto Lupus Clinic. Disease activity was determined using the SLE disease activity index 2000 (SLEDAI-2K) (25), with the clinical SLEDAI-2K (cSLEDAI) being calculated by omitting the contribution of hypocomplementemia and anti-dsDNA positivity from the total score. Only patients with a recent flare (change in cSLEDAI ≥1 within the last month prompting a change in therapy) or prolonged quiescence (cSLEDAI =0 for >1 year without escalation of therapy and taking <10 mg/day prednisone at the time of recruitment) were recruited from the clinic for the study. Sex-matched ANA-negative (by immunofluorescence) and specific antibody- (by Bioplex ANA screen) negative healthy controls (HC) were recruited from hospital/laboratory personnel. All patients were followed longitudinally, with the clinical data being collected using a standardized protocol. Peripheral blood mononuclear cells (PBMCs) were archived at the time of recruitment and approximately every 6 months thereafter, COVID permitting. The study was approved by the University Health Network (UHN) Research Ethics Board and all participants signed informed consent.

### Cell staining

Frozen archived PBMCs from SLE patients and HCs were thawed (post-thaw viability > 90%) and 2 million cells were then stained with a panel of metal tagged antibodies (**Supplementary Table 1**), as previously published (26, 27). Briefly, thawed cells were washed with phosphate-buffered saline (PBS) and stained with 1μM viability dye cisplatin (Standard Biotools). The cells were then fixed in fixation buffer (eBioscience), permeabilized, and barcoded using the Cell-ID 20-Plex Pd Barcoding Kit (Standard Biotools). The individual samples were pooled, incubated with Fc receptor block (ThermoFisher) for 10 minutes, and stained with an extracellular antibody cocktail at room temperature for 30 minutes. Following washing, cells were incubated in intracellular antibody cocktail in permeabilization buffer (eBioscience) at room temperature for 30 minutes. Finally, they were washed and incubated in 100nM Cell-ID Intercalator-Ir (Standard Biotools) at 4 °C overnight to aid in identifying cells later. Acquisition was done using a Helios mass cytometer (Standard Biotools) at the Hospital for Sick Children’s Mass Cytometry Facility. PBMCs from patients with multiple visits were stained and processed in the same batch. Age- and sex-matched HCs were included in each CyTOF run to allow normalization for variability.

### Measurement and calculation of ISG scores

Total RNA was isolated from whole peripheral blood archived in Tempus™ tubes (ThermoFisher) and gene expression quantified using a custom NanoString (NanoString Technologies), as previously reported (28). Three sets of ISGs were examined that were representative of the M1.2 (ISG.1), M3.4 (ISG.2) and M5.12 (ISG.3) gene clusters previously identified on transcriptomic profiling of SLE patients (29). Log_2_ normalized expression levels of the ISGs were summed to generate composite scores for each cluster.

### IFN incubation assay

Freshly isolated PBMCs from ANA-negative healthy individuals (n=4) were seeded in 24-well ultra-low attachment plates (1 x 10^6^ cells per well in a 1 ml volume). Cells were either incubated in media alone or stimulated with 250 or 1000 U/ml IFNα, IFNβ or IFNγ (PBL biosciences), for 18 or 24 hours. Monensin (1X, Biolegend) and Brefeldin A (1µg/ml, Biolegend) were added to each well for the last 2 hours of incubation. The cells were then stained for CyTOF, as outlined previously.

### Statistical analysis

CyTOF data files were pre-processed to exclude beads, debris, doublets, and dead cells. Viable CD45+ cells were selected for downstream analysis, as shown in the full pre-processing gating hierarchy in **Supplementary Figure 2**. Marker expression values were arc.sinh-transformed prior to downstream analysis. To determine the phenotypes of cell subsets, R (v4.4.1) was used to normalize phenotyping markers with CyCombine (v0.2.5) to account for batch-to-batch variation. Graph-based clustering (k-nearest neighbors = 25) was then performed on pooled CD45+ cells from all participants using FastPhenograph (v0.0.6), and UMAP figures were generated using UMAP (v0.3.10), as previously described (26, 27). Cell subsets were then identified using heatmaps showing relative expression of cell lineage defining markers, as shown in **Supplementary Figure 1** with the key markers used to identify each population listed in **Supplementary Table 2**, and confirmed by manual gating in FlowJo (v10.0) using previously described gating strategies (28) (**Supplementary Figures 3**–**5**). For the CyTOF results following *in-vitro* IFN incubation, phenotyping was done using FlowJo. Trafficking markers and ISPs were normalized separately on a per-phenotype basis to account for batch effects by normalizing the median marker expression in each SLE sample to the median expression of the corresponding cell population in HCs analyzed within the same experimental round. For each cell, the average ISP score was calculated using the mean expression of the six ISPs (ISG15, IFIT3, MX1, PKR, IRF7, and BST2). For each immune cell population within each sample, the median of these average ISP scores was then calculated, and the normalized ISP score was generated by dividing this value by the median average ISP score of the corresponding immune cell population in the HC samples analyzed in the same CyTOF round.

Statistical comparisons were performed using Student’s t-test or Wilcoxon rank-sum test for two-group comparisons, depending on the data distribution. Demographic and treatment characteristics of the study participants are summarized in **Supplementary Table 3**, and statistical comparisons of these characteristics between patient groups are provided in **Supplementary Table 4**. Categorical variables were compared using Fisher’s exact test, while prednisone dose was compared using the Wilcoxon rank-sum test. For multiple group comparisons, ANOVA or Kruskal-Wallis tests were used followed by *post-hoc* pairwise comparisons with Bonferroni correction. In some cases, after confirming normality using the Shapiro-Wilk test, statistical significance was determined using Student’s t-test with Holm correction for multiple comparisons. Correlations were assessed using Spearman correlation coefficients with Benjamini-Hochberg correction as appropriate. Correlation matrices were generated using the R packages Hmisc (v5.2.3) and corrplot (v0.92). Logistic regression models were generated using the R stats package to assess associations between baseline variables and subsequent flare status. Receiver operating characteristic (ROC) curves and area under the curve (AUC) values were generated using the R package pROC (v1.18.5). For analyses involving multiple cell populations or markers, Benjamini-Hochberg false discovery rate correction was applied as indicated. p values less than 0.05 were considered statistically significant.

Differential cell abundance and differential state analyses between patient groups were performed using the R package diffcyt (v1.24.0) (30) and visualized with ggplot2 (v3.5.1). Differential abundance was assessed using the testDA_edgeR method, which uses edgeR-based generalized linear models to test for differences in cell population counts between groups. Differential state analyses were performed using the testDS_limma method, which uses limma-based linear models to test for differences in median marker expression within individual cell populations between groups. For comparisons of normalized ISP scores, individual ISPs, and trafficking molecules, each marker was treated as a state marker, and differences in expression were tested independently within each of the 29 immune cell populations. For analyses adjusting for ethnicity, ethnicity was included as a categorical covariate in the design matrix in addition to the patient group being compared (**Supplementary Table 5**). For longitudinal or paired comparisons, patient identity was included in the statistical design to account for repeated measurements from the same individual. Heatmaps were generated using pheatmap (v1.0.12) with hierarchical clustering.

## Results

### Establishment of a panel of antibodies that enables examination of ISP expression at a single cell level using CyTOF

Previous studies suggest that there are three distinct clusters of ISGs found in the peripheral blood of SLE patients that may represent genes induced by distinct IFNs or different strengths of IFN signals (29, 31). Therefore, to investigate ISG expression at a single cell level in SLE patients, we used antibodies directed against six ISPs encoded by genes representative of each of the clusters: M1.2 (ISG15, IFIT3, MX1), M3.4 (PKR, IRF7), and M5.12 (BST2) (29). These antibodies were incorporated into a 41-marker CyTOF panel (**Supplementary Table 1**) that enabled identification of diverse immune cell populations (**Supplementary Figures 1**, **3**–**5**) previously implicated in the pathogenesis of SLE, together with cellular markers associated with activation, proliferation, and trafficking.

The panel was then used to examine freshly thawed PBMCs from 46 SLE patients with a recent flare, 24 quiescent SLE patients, and 18 ANA-negative HCs, as well as follow-up samples obtained at 6, 12, or 36 months from 20 of the SLE patients, with demographics shown in **Supplementary Table 3**. Twenty-nine distinct immune populations were identified using an un-supervised clustering approach (**Figure 1A**). Heatmaps showing the expression of lineage-defining markers for each cluster are shown in **Supplementary Figure 1**, with the key markers used to identify each population listed in **Supplementary Table 2** and the manual gating used to confirm identity of the clusters shown in **Supplementary Figures 3**–**5**. The populations identified included seven B cell subsets (transitional, naïve, activated naïve, unswitched memory (USM), age-associated B cells (ABC), switched memory (SM), and plasmablast (PB)), twelve T cell subsets (naïve CD4+, naïve CD8+, naïve double negative (DN), double positive (DP), activated CD4+/CD8+, effector CD8+, effector DN, memory CD4+, memory CD8+, T follicular helper (Tfh), T peripheral helper (Tph), and CD4+/CD8+ regulatory T (Treg)), four monocyte subsets (classical monocytes, including three groups based on their activation level, with 1 the lowest and 3 the highest, and non-classical/intermediate monocytes), two NK cell subsets (CD16+ and CD16-), three dendritic cell (DC) subsets (plasmacytoid DC (pDC), activated pDC, and myeloid DC (mDC)), and low-density granulocytes (LDG).

**Figure 1:**
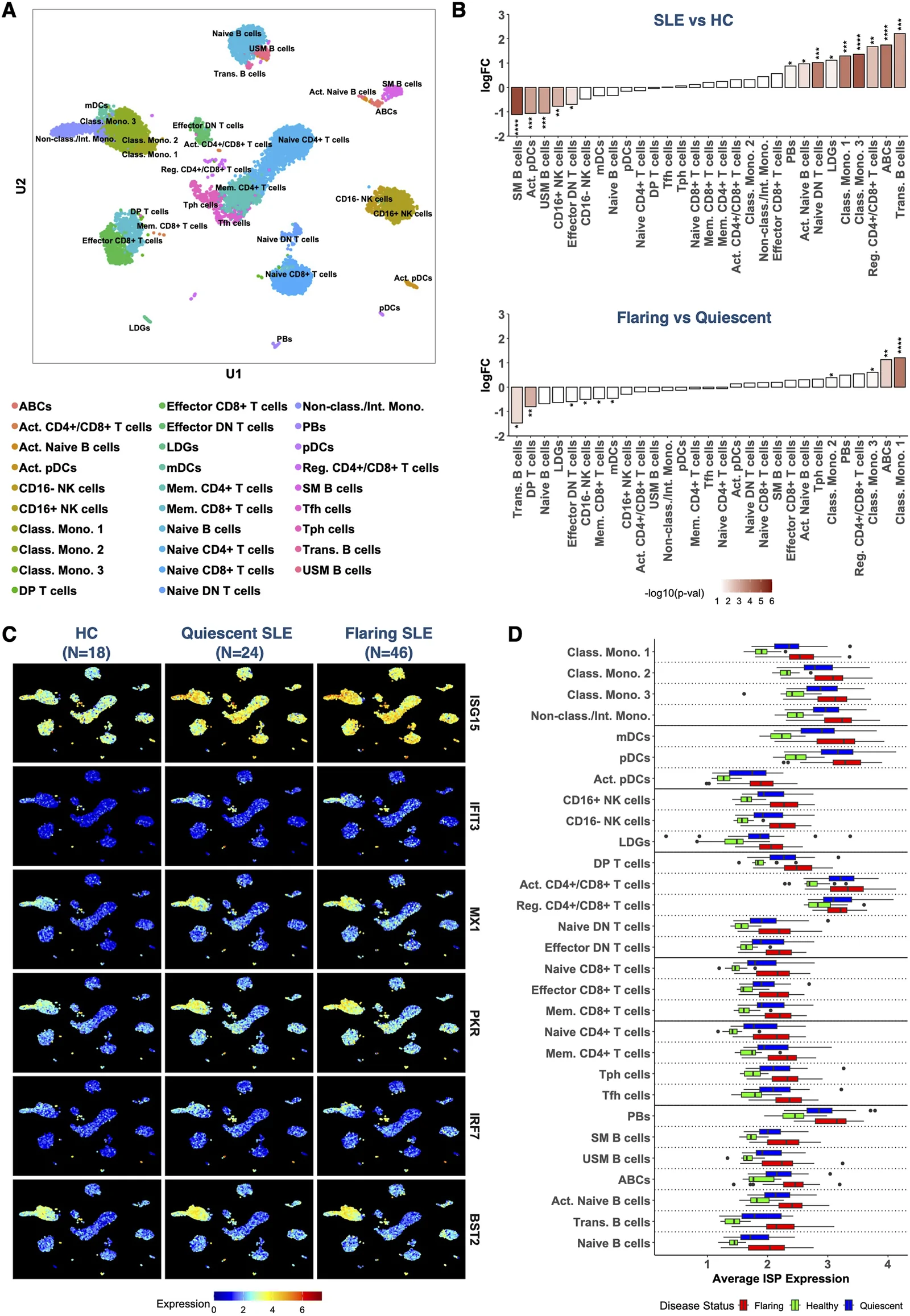
Identification of 29 distinct cell populations and their ISP levels using CyTOF. **(A)** UMAP of the pooled PBMCs from SLE patients and healthy controls. Phenotypes are annotated by the colours shown in the adjacent legend. **(B)** Differences in immune cell population abundance comparing SLE patients with healthy controls or flaring with quiescent SLE patients. Bars above 0 indicate increased abundance in SLE patients compared to healthy controls, or in flaring compared to quiescent patients. Differential abundance was assessed using diffcyt-DA-edgeR, with log2 fold changes estimated from empirical Bayes-moderated models and significance determined using FDR-corrected p values. Statistical significance is denoted by both colour and asterisks, with *p ≤ 0.05, **p ≤ 0.01, ***p ≤ 0.001, and ****p ≤ 0.0001. **(C)** UMAP showing the expression of the six ISPs in healthy controls, quiescent patients and flaring patients. ISPs shown include ISG15, IFIT3, MX1, PKR, IRF7, and BST2. **(D)** Mean expression levels of the six ISPs from **Figure 1C** for each cell subset in healthy controls, quiescent patients and flaring patients.

Comparison of demographic and treatment characteristics between the flaring and quiescent groups showed that ethnicity was the only variable that differed significantly between the groups (Fisher’s exact test, p=0.0449; **Supplementary Table 4**). We therefore performed additional analyses adjusting for ethnicity to determine whether differences between patient groups were independent of this demographic imbalance (**Supplementary Table 5**). Analysis of cell subset abundance between SLE patients at the initial visit and HCs revealed significantly higher frequencies of transitional B cells, ABCs, regulatory CD4+/CD8+ T cells, classical monocytes groups 1 and 3, LDGs, naïve DN T cells, activated naïve B cells, and PBs in SLE patients than in HCs (**Figure 1B**). Of these populations, only the classical monocyte populations and ABCs were significantly more abundant in flaring than quiescent SLE patients. Across all 29 populations, 19 showed significant differences in abundance between SLE patients and HCs before adjustment for ethnicity, of which 12 remained significant following adjustment: ABCs, activated pDCs, CD16- NK cells, CD16+ NK cells, classical monocyte groups 1 and 3, effector CD8+ T cells, naïve DN T cells, regulatory CD4+/CD8+ T cells, SM B cells, transitional B cells, and USM B cells. Similarly, 7 populations differed significantly between flaring and quiescent SLE patients before adjustment, with 5 remaining significant after adjustment for ethnicity: transitional B cells, naïve B cells, mDCs, CD16- NK cells, and classical monocyte group 1 (**Supplementary Table 5**). We have previously extensively characterized the immunophenotype of the patients in the current cohort using flow cytometry (28). While the immune cell populations examined using unbiased clustering did not precisely match those investigated in this previous study, comparisons of cellular abundance between SLE patients and HCs, or between flaring versus quiescent patients, recapitulated many of the findings of that study, suggesting that both approaches identify comparable changes between subject groups.

UMAP results showing expression of each of the six ISPs in pooled PBMCs from HCs, quiescent, and flaring SLE patients at the initial visit are shown in **Figure 1C**. The average levels of ISP expression, generated by calculating the mean of the six ISPs in each cell, appeared lowest in HCs, intermediate in quiescent patients, and highest in flaring patients across all populations, as shown descriptively in **Figure 1D**, with statistical comparisons of normalized ISP scores between flaring and quiescent SLE patients presented in **Figure 2C**. Notably, there was considerable variability in ISP expression between cell types, with monocytes, DCs, activated and regulatory CD4+/CD8+ T cells, and PBs showing higher levels than other cell populations, regardless of subject group.

**Figure 2:**
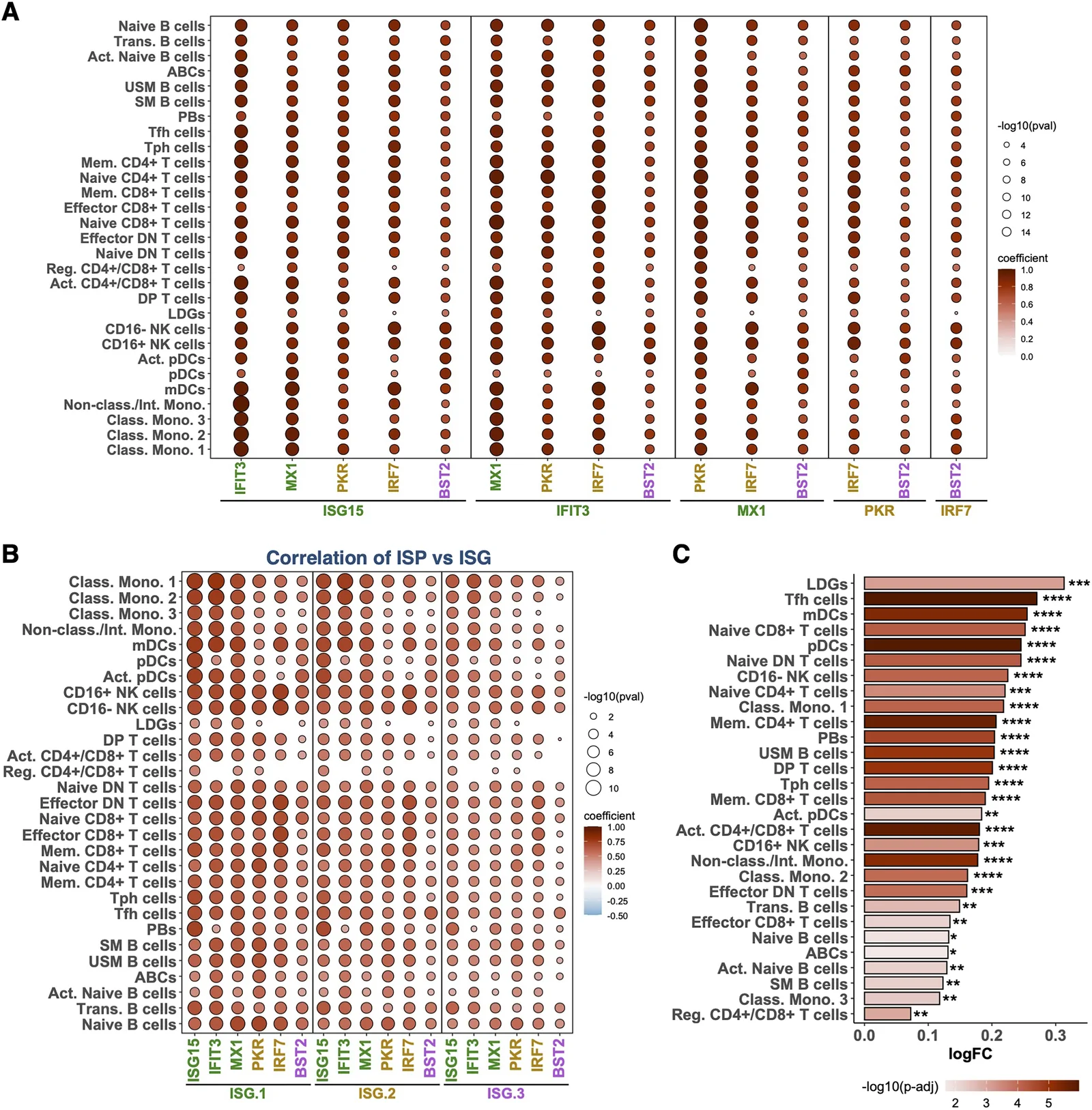
Evaluation of ISPs as surrogate markers of the IFN signature. **(A)** Single-cell correlation of each ISP with the others. Spearman R values are denoted by colour, and p values are represented by size. Only populations with significant Spearman correlations (p ≤ 0.05) are shown. **(B)** Correlation matrix of global ISG.1, ISG.2 and ISG.3 scores versus the average expression of the six ISPs in each cell subset. **(C)** Waterfall plot showing differential levels of the composite ISP score between flaring and quiescent patients, with bars to the right of 0 indicating increased expression in flaring patients. The ISP score was calculated based on the normalized average expression of the six ISPs measured: ISG15, IRF7, MX1, PKR, IFIT3, and BST2. Differential expression was assessed using diffcyt-DS-edgeR, with log2 fold changes estimated from empirical Bayes-moderated models and significance determined using FDR-corrected p values. Statistical significance is denoted by both colour and asterisks, with *p ≤ 0.05, **p ≤ 0.01, ***p ≤ 0.001, and ****p ≤ 0.0001.

### The levels of ISP expression in most immune populations correlate strongly with peripheral blood ISG scores and are higher in flaring than quiescent SLE patients

Incubation of freshly isolated PBMCs from ANA-negative HCs with IFNα, IFNβ, or IFNγ confirmed that all ISPs were strongly induced in all immune populations by IFNα and IFNβ (**Supplementary Figure 6A**). In contrast, minimal induction of the ISPs by IFNγ was seen, except for IFIT3, which was induced in classical monocytes and CD14+ DCs. The relative levels of ISPs in each cell population following incubation with IFNα or IFNβ mirrored those seen for ex-vivo cells, suggesting that the differences seen between ex-vivo populations reflect differences in the capacity of their cells to respond to IFNs (**Supplementary Figure 6B**).

To enable comparisons between patients across different CyTOF runs, the levels of non-phenotyping proteins within the immune populations of each patient were averaged and then normalized to the levels seen in the HC immune populations included in the same round. Very strong correlations were found for most cell populations between the normalized individual ISP levels with each other (**Figure 2A**), and with three whole blood ISG expression scores calculated from representative genes from the M1.2, M3.4, and M5.12 gene clusters (29), referred to here as ISG.1, ISG.2, and ISG.3, respectively (**Figure 2B**). In general, the correlations between the six ISPs and ISG scores were strongest with ISG.1 and weakest with ISG.3 regardless of which cluster of genes encoded the ISP (i.e., ISG.1, ISG.2, or ISG.3).

Since the normalized individual ISPs were strongly and roughly equivalently correlated with global ISG expression, a composite normalized ISP score was generated for each patient’s cell populations (see Materials and Methods). For all cell types, this normalized ISP score was significantly higher in flaring than quiescent SLE patients (**Figure 2C**). These findings are consistent with those of previous studies indicating that flaring patients are more likely to have higher levels of ISG expression than quiescent patients (10–12) and indicate that the global peripheral blood ISG score, regardless of which genes are included, closely mirrors the mean levels of ISP expression in the majority of immune cell populations. To determine whether these differences were independent of the racial/ethnic composition of the groups, the analysis was repeated with ethnicity included as a categorical covariate. Normalized ISP scores remained higher in flaring patients across all 29 populations, with differences remaining significant in 27 of 29 populations after adjustment for ethnicity and Benjamini-Hochberg correction for multiple comparisons (**Supplementary Table 5**). Naïve B cells and classical monocyte group 3 were the only populations that did not remain significant following adjustment.

### Heterogeneous levels of ISP expression are seen within individual cell populations

Consistent with previous reports suggesting that distinct subsets of cells within some immune populations have higher levels of ISG expression (23), considerable variability was seen in the levels of ISP expression in several immune populations in the pooled UMAP (**Figure 1C**). This led us to assess whether this variability reflected heterogeneity in ISP expression between patients or within patients. To address this question, we examined expression of ISPs at a single cell level in the immune populations of individual patients. Representative results from SLE patients with high (top 15%) and low (bottom 15%) ISG scores, together with HCs, are shown in **Supplementary Figure 7A**.

For the ABC and Tph cell populations, there was heterogeneity in the levels of the various ISPs expressed, and as expected based upon their correlation with each other following IFN stimulation, the ISPs tended to be coordinately expressed (**Supplementary Figure 7Ai, ii**). As shown in the histograms at the bottom of each heatmap, regardless of the subject group, the average expression of the six proteins demonstrated a roughly unimodal distribution that was not normally distributed, with evidence in some subjects of a second peak or shoulder of cells with high ISP expression. In contrast, classical monocyte 3 and mDCs had a broader distribution of ISP expression, with a more distinct pattern of high and low ISP-expressing cells in both ISG^hi^ and ISG^low^ patients (**Supplementary Figure 7Aiii, iv**). These findings indicated that some of the heterogeneity arises from differences within each patient’s immune populations.

To determine whether this within-patient variability was an intrinsic property of the response of these cell populations to Type I IFNs, we examined within-population variability in the upregulation of the individual ISPs in HC PBMCs incubated with IFNα or IFNβ *in-vitro*. Apart from intermediate/non-classical monocytes and CD16- NK cells, incubation with these cytokines induced a unimodal distribution of upregulated expression (**Supplementary Figure 7B**). Furthermore, while a bimodal pattern of expression was seen for intermediate and non-classical monocytes, as well as CD16- NK cells *in-vitro*, this pattern did not recapitulate that seen *in-vivo* for these populations. Thus, the within-population ISP variability likely arises from variable exposure of the cells to IFN and/or other signals that alter their capacity to respond to IFNs *in-vivo*.

### Differences in trafficking and activation could explain heterogeneity in the ability of cells to respond to IFN

One potential mechanism by which cells could be exposed to different concentrations of IFNs or other signals that could alter their capacity to respond to IFN is through trafficking to inflamed tissues. To explore this possibility, we assessed the expression of some representative trafficking markers across our cell populations, including β7, an integrin involved in homing to and retention in the small intestine (32); CCR9, a chemokine receptor associated with small intestine tropism (33); CLA, a skin-homing molecule (34); and L-selectin, which facilitates lymph node homing (35). Representative results for switched memory B and Tph cells are shown in **Figure 3**. As previously reported, only memory B and T cells expressed appreciable levels of trafficking molecules (**Figure 3A**), with most of the cells expressing these molecules being in the ISG^hi^ sub-population (**Figure 3B**). In contrast, myeloid cells displayed little to no expression of these markers (**Supplementary Figure 8A**). To confirm whether similar findings extended to other memory T and B cell populations, expression of the trafficking molecules was contrasted between the top 10% and remaining 90% of ISP-expressing cells in individual patients. As shown in **Figure 3C**, for all memory populations there was significantly higher expression of most trafficking molecules in the ISP^hi^ sub-population. In general, the trafficking molecules tended to be expressed in distinct cells within each immune population, reflecting their distinct tissue localization patterns (representative results shown in **Figure 3D**, with correlations for the top 10% of ISP^hi^ cells shown for all memory populations in **Figure 3E**). CCR9 and β7 were the only exception to this pattern, which were weakly but consistently correlated in keeping with their shared role in gut homing.

**Figure 3:**
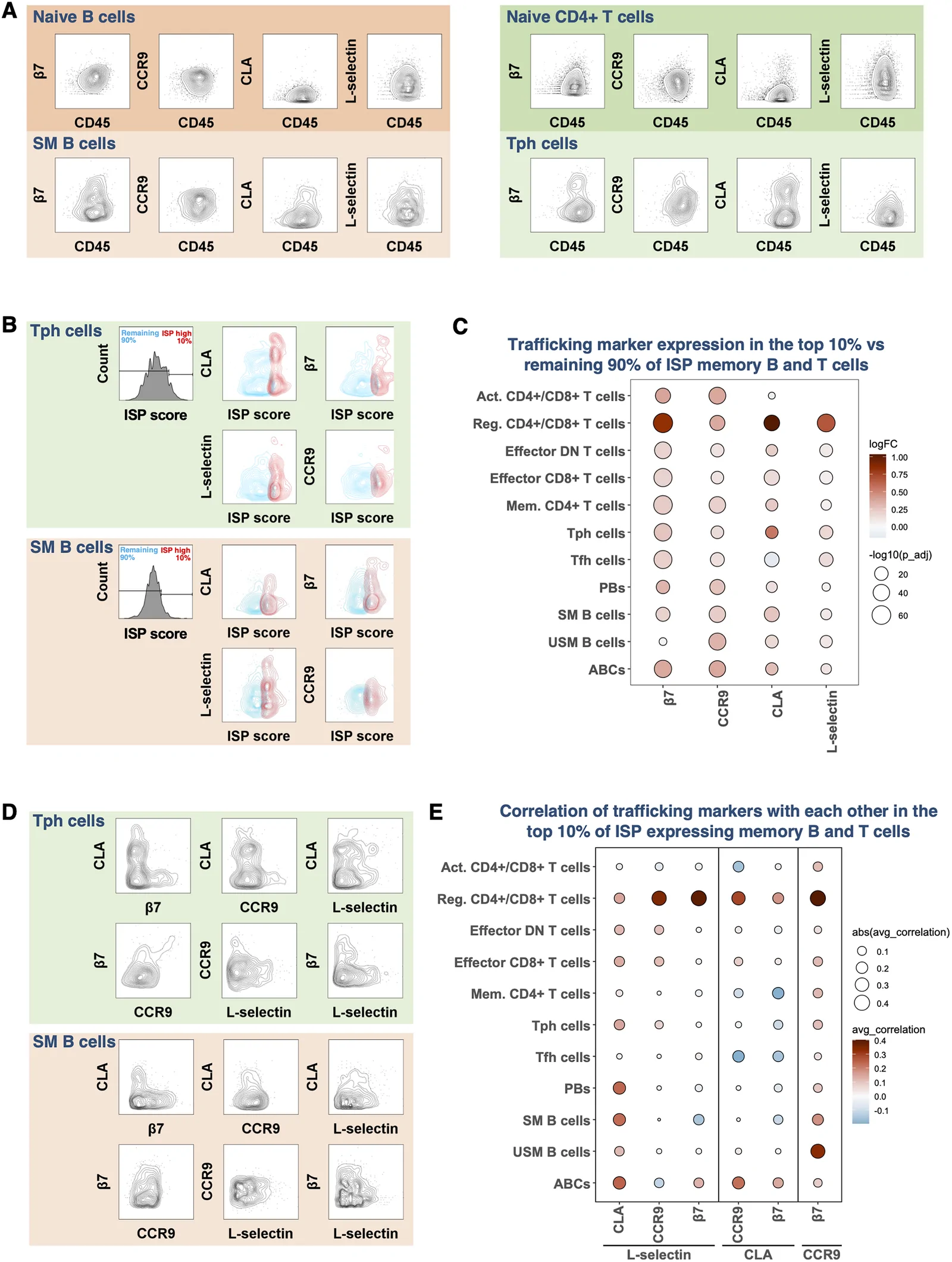
Trafficking molecule expression patterns and association with ISP levels **(A)** Representative flow plots showing expression of the trafficking molecules β7, CCR9, CLA, and L-selectin in naïve and memory B and T cell subsets from a representative SLE patient. Memory cell subsets shown are SM B and Tph cells. **(B)** Expression of trafficking molecules in the top 10% and remaining 90% of ISP-expressing cells within Tph and SM B cells. ISP-high cells are shown in red, while the remaining cells are shown in blue. **(C)** Comparison of trafficking molecule expression in the top 10% versus remaining 90% of ISP-expressing cells within each memory lymphoid cell population from individual patients. Differential expression was assessed using diffcyt-DS-edgeR, with log2 fold changes estimated from empirical Bayes-moderated models and significance determined using FDR-corrected p values. Log fold change values are denoted by colour, and adjusted p values are denoted by dot size. **(D)** Representative flow plots showing coordinated expression patterns of trafficking molecules in Tph and SM B cells from a representative SLE patient. Each trafficking molecule is shown relative to the other trafficking molecules. **(E)** Single-cell correlation of each trafficking molecule with the others across memory lymphoid cell subsets in the top 10% of ISP-expressing cells. Spearman R values are denoted by colour and size.

To determine whether IFNs directly induced these trafficking molecules, their expression was assessed in HC cells following *in-vitro* IFN stimulation (**Supplementary Figure 8B**). Only β7 was moderately to strongly induced by IFNα and IFNβ, while L-selectin showed weak induction. In contrast, CCR9 and CLA were not significantly induced by any of the IFNs, except for transient CLA upregulation in DCs at 18 hours. These findings suggest that the higher expression of trafficking molecules in ISP^hi^, as compared to ISP^low^ cells, does not result from induction by IFNs. Taken together, the results suggest that trafficking of cell populations to inflamed tissues may explain some of the heterogeneity in ISP expression seen in the PBMCs of SLE patients.

### CLA expression in Tph cells associates with skin involvement during flare

To further investigate the association between cells with high trafficking marker expression and tissue inflammation, we focused on CLA^hi^ cells, which have been previously found to be increased in patients with various inflammatory skin conditions (36). Since Tph cells have been identified in the skin of SLE patients, we examined whether the proportion of CLA^hi^ cells varied over time and whether it was associated with skin involvement. Flow plots from a representative flaring SLE patient with a skin rash at baseline that resolved during follow-up are shown in **Figure 4A**. Among flaring patients at baseline, those with a skin rash had significantly higher proportions of CLA^hi^ Tph cells than those without a skin rash (median 3.20% vs 0.96%; Wilcoxon rank-sum test, p = 0.021; **Figure 4B**). Consistent with this association, all but one of the patients with a skin rash at baseline had resolution of the rash by the 6-month follow-up, and CLA^hi^ Tph cell frequencies significantly decreased over this period (Wilcoxon signed-rank test, p = 0.030; **Figure 4C**). The patient with persistent skin rash had relatively low CLA^hi^ Tph cell frequencies at both baseline (0.72%) and 6 months (0.87%). Among the remaining patients, the decrease in CLA^hi^ Tph cells accompanied resolution of the skin rash, despite continued disease activity in other organ systems in a subset of patients. In contrast, CLA^hi^ Tph cell frequencies did not significantly change from baseline to 6 months in patients without a skin rash at baseline (p = 1.00). Notably, one patient without a skin rash at baseline showed an increase in CLA^hi^ Tph cell frequency from 0.60% at baseline to 6.58% at 6 months and subsequently developed a skin rash between the 6 month and 1 year visits, maintaining an elevated CLA^hi^ Tph cell frequency of 3.43% at the 1-year follow-up. These findings suggest that, during periods of disease activity, some circulating ISP^hi^ cells may reflect populations that have trafficked through inflamed tissues and may therefore provide information about organ-specific inflammation.

**Figure 4:**
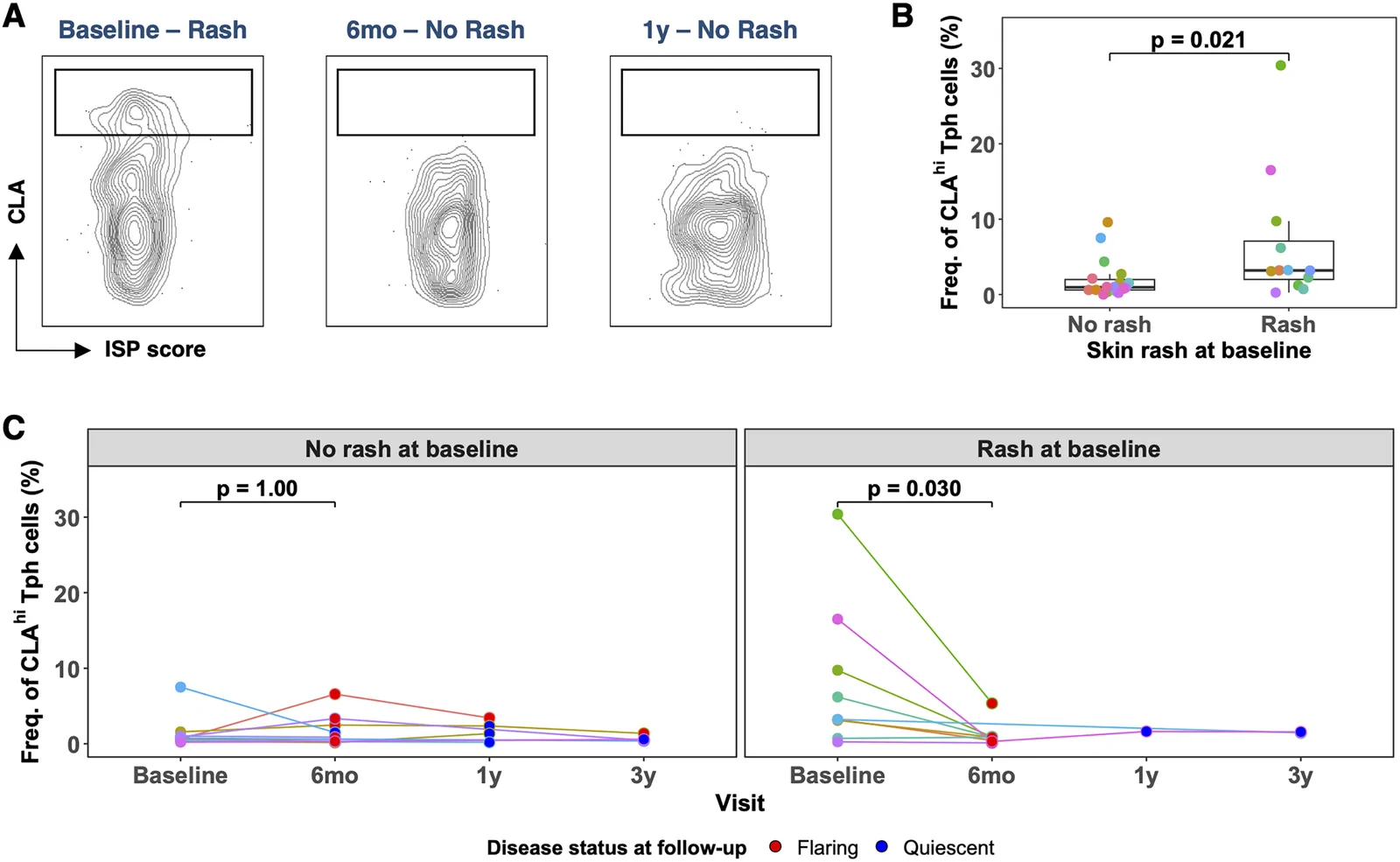
CLA expression in Tph cells and association with skin involvement. **(A)** Representative flow plots showing the gating strategy used to identify CLA^hi^ Tph cells in a flaring SLE patient with a skin rash at baseline that resolved at subsequent follow-up visits. **(B)** Frequency of CLA^hi^ Tph cells at baseline in flaring SLE patients with or without skin rash. Individual patients are represented by coloured dots. CLA^hi^ Tph cell frequencies were compared using a Wilcoxon rank-sum test. **(C)** Longitudinal frequency of CLA^hi^ Tph cells in SLE patients who were flaring at baseline, separated according to the presence or absence of skin rash at first visit. Individual patients are represented by coloured dots at baseline, and corresponding follow-up visits are connected by a line of the same colour. Follow-up disease status was determined based on cSLEDAI, with flaring patients shown in red and quiescent patients shown in blue. Among patients with skin rash at baseline, only one continued to have a skin rash at the 6-month follow-up. Baseline and 6-month frequencies were compared within each group using a paired Wilcoxon signed-rank test.

### Longitudinal changes in ISP expression and their association with changes in disease activity

Prior longitudinal studies from our and other groups have shown that ISG expression remains relatively stable over time in most SLE patients (12, 22). However, single cell RNA-Seq data suggest that this lack of variation may not be representative of all immune populations (23). To investigate how ISP expression changes in various immune populations over time following treatment, follow-up samples from 20 SLE patients obtained at 6, 12 or 36 months were run in tandem with their baseline samples. These patients were either flaring or quiescent at initial visit (demographic data shown in **Supplementary Table 3**). As expected, for most patients active at baseline, the cSLEDAI consistently decreased after 6 months in response to treatment, whereas most quiescent patients remained stable with a cSLEDAI of 0 (**Figure 5A**). However, when exploring the trends in ISP score over the same time period, we found that while the ISP score did not change dramatically from baseline at the 6-month or 1-year follow-up visits, patients who were flaring at baseline had significant reductions in the ISP scores of most of their activated, effector, and memory T cell subsets, and activated innate immune subsets, including activated pDCs, non-class/intermediate monocytes, and classical monocyte group 3, at 3-year follow-up (**Figure 5B**; **Supplementary Figure 9A**). Notably, B cell subsets did not show a similar reduction in ISP expression over the same period and quiescent patients maintained largely stable ISP scores in all immune populations.

**Figure 5:**
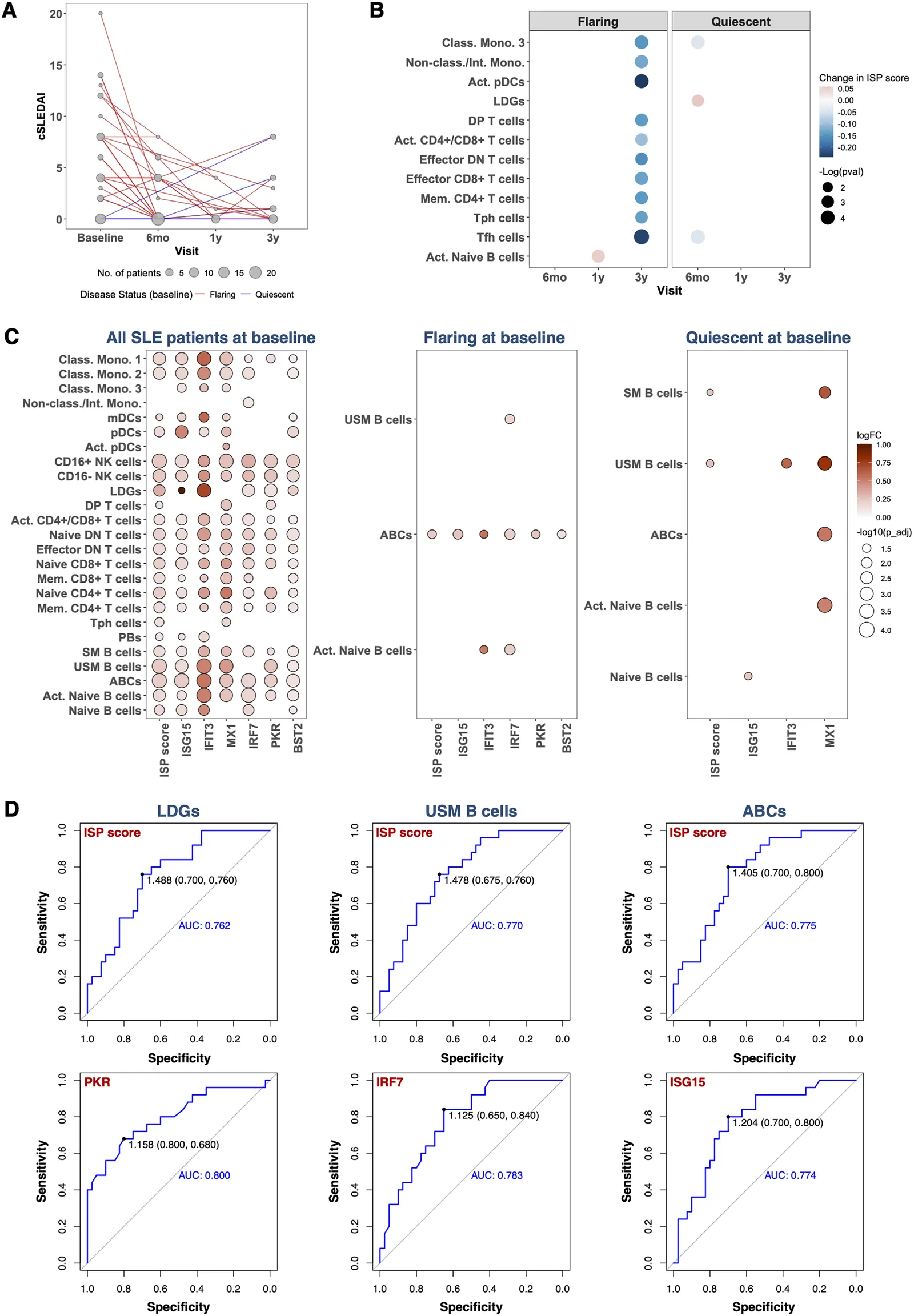
Changes in ISP scores over time and association with longitudinal disease activity **(A)** cSLEDAI of patients with longitudinally acquired samples at 6-month, 1-year, and 3-year follow-up visits. Lines are coloured according to whether patients were flaring or quiescent at baseline, with flaring patients shown in red and quiescent patients shown in blue. Dot size denotes the number of patients at each visit with the same cSLEDAI score. **(B)** Dot plot showing change in ISP score over time relative to the initial visit in patients with multiple visits. An increase in ISP score between the first and longitudinal visit is denoted in red, while a reduction is denoted in blue. Statistical significance was determined using the Mann Whitney U test. Dot size denotes p value, and only significant changes are shown. **(C)** Differences in baseline ISP levels between SLE patients who had disease flare versus disease quiescence at 1-year follow-up. Patients were first assessed together and then stratified by disease status at baseline. When patients were stratified by baseline disease status, only B cell subsets had significantly different baseline ISP scores, with higher ISP scores observed in patients who flared 1 year later. Differential expression was assessed using diffcyt-DS-edgeR, with log2 fold changes estimated from empirical Bayes-moderated models and significance determined using FDR-corrected p values. Statistical significance is denoted by both colour and asterisks, with *p ≤ 0.05, **p ≤ 0.01, ***p ≤ 0.001, and ****p ≤ 0.0001. **(D)** ROC curves showing the best predictors of longitudinal disease flare 1 year later. ISP expression levels in LDGs, USM B cells, and ABCs had the highest area under the curve values, as shown on the graphs.

Elevated levels of ISG expression and/or IFNα at baseline are associated with an increased risk of subsequent flare. We therefore questioned whether this was also the case for elevated levels of ISP expression at baseline. As shown in **Figure 5C**, when all SLE patients were included regardless of baseline clinical status, elevated levels of the individual ISPs and the global ISP score in most immune populations were moderately to strongly associated with subsequent disease activity 1 year later. However, when patients were stratified based on whether they were active or quiescent at baseline, only elevated ISP expression in B cell subsets was associated with disease activity 1 year later. This finding suggests that immune mechanisms associated with the effect of IFN on B cells may play an important role in promoting disease activity in SLE.

To explore further the potential for measurement of individual ISPs to predict disease activity 1 year later in SLE patients, we conducted a ROC analysis across all cell subsets and ISPs (**Supplementary Figure 9B**). We found that, of all possible combinations of cell subsets and ISP measurements, elevations in the ISP expression within LDGs and B cell subsets were the most predictive of future disease activity (**Figure 5D**). Specifically, PKR in LDGs had an AUC of 0.8, IRF7 in USM B cells had an AUC of 0.783, and ISG15 in ABCs had an AUC of 0.774. Taken together, these results indicate that ISP levels in B cell subsets demonstrate minimal fluctuation with changes in disease activity and were the strongest predictors of subsequent disease activity.

## Discussion

In this study, we leveraged longitudinal samples from SLE patients to highlight the interplay between IFN-driven immune cell activation, cellular trafficking, and disease activity over time using ISP expression as a surrogate marker to quantify IFN signaling in cell subsets (26). We showed that the six ISPs used in this study are coordinately expressed, both *in-vivo* and *in-vitro*, and demonstrate a moderate to strong correlation at the cell population level with global ISG signatures, supporting their use as cellular readouts of systemic IFN activity. In all populations, the six ISPs were most strongly associated with ISG.1 (M1.2) and the least associated with ISG.3 (M5.12). However, in innate immune populations, ISPs encoded by ISG.1 (M1.2) genes were strongly associated with all three ISG scores, whereas for the majority of B and T cell populations, ISPs encoded by both ISG.1 (M1.2) and ISG.2 (M3.4) genes were strongly and roughly equivalently associated with the ISGs. These findings tended to parallel those seen *in-vitro*. Consequently, the differences between ISG.1 (M1.2) and ISG.2 (M3.4) genes may not only reflect greater responsiveness of ISG.1 (M1.2) genes to IFNα and/or low concentrations of Type I IFNs, as previously suggested (31), but may also reflect their differential expression across immune populations. Notably, the correlation between ISP expression and ISG.3 (M5.12) was the poorest of the three scores. It has been previously reported that the M5.12 gene cluster is enriched for genes that are induced by IFNγ (31). As reported by others (37) and confirmed here, none of the ISPs in our study were strongly induced by IFNγ, including BST2, which is encoded by an M5.12 gene. This may provide an explanation for the poorer correlation with this gene cluster. Taken together, these data suggest that expression of the six ISPs assessed in this study are excellent surrogate measures of Type I, but likely not Type II, IFN-induced gene expression.

The significant within-patient variability in ISP expression within individual immune populations of SLE patients emphasizes the importance of studying the IFN signature at a single-cell level. Given that incubation of HC immune populations with Type I IFNs resulted in a homogeneous response, we postulated that the differential expression of ISPs in circulating immune cells was due to variable IFN exposure, potentially linked to cellular trafficking. Previous experiments indicate that many sites of tissue inflammation in SLE have high expression of IFNs, including the lymph nodes, kidneys, intestinal tract, and skin (38–41). We provide several lines of evidence consistent with the concept that ISP^hi^ cells may have trafficked to these sites. Firstly, elevated levels of trafficking markers were enriched on the surface of memory T and B cell populations, and particularly within the ISP^hi^ subset of these cells. Secondly, trafficking markers associated with different tissue compartments were largely expressed exclusively, whereas the two gut-homing markers were to some extent coordinately expressed. Thirdly, except for β7, the trafficking markers were generally not strongly induced by Type I IFNs. In further support of this concept, flaring SLE patients with skin rash had significantly higher proportions of CLA^hi^ Tph cells than those without skin rash. Among patients with skin rash at baseline, all but one had resolution of the rash by the 6-month follow-up, and CLA^hi^ Tph cell frequencies significantly decreased over the same period, whereas no significant change was observed in patients without skin rash. Overall, the association of CLA^hi^ Tph cells with active skin involvement and their decline following resolution of the rash is consistent with these cells trafficking through inflamed skin.

In addition to generalized induction of β7, temporal analysis demonstrated transient CLA upregulation in DCs following Type I IFN exposure, followed by downregulation with prolonged stimulation. This finding suggests that DCs may transiently acquire skin-homing potential when they are first exposed to Type I IFNs. Consistent with this, we have observed a negative association between mDC frequency in the blood and their IFN signature (data not shown). In contrast, CLA expression in Tfh cells was downregulated following IFN exposure *in-vitro* and was lower in ISP^hi^ compared to ISP^low^ cells, suggesting that IFN may influence Tfh trafficking patterns by directing them away from the skin, and toward lymph nodes and mucosal interfaces. Collectively, these findings indicate a complex interplay between IFN and cellular trafficking in SLE.

As reported previously for the global peripheral blood IFN signature (12, 22), ISP expression remained stable in flaring patients at 6 and 12 months following treatment despite normalization of clinical disease activity. However, at 3 years, significantly reduced ISP expression was seen in several immune populations, including all activated/effector T cell subsets, Tph and Tfh cells, and several innate immune populations whose expansion is associated with chronic TLR activation. These findings indicate that the IFN signature in some immune populations fluctuates with disease activity, but the kinetics of this fluctuation are significantly delayed. One of the factors that may contribute to this delay is our previous observation that several immune populations that appear to be depleted from the peripheral blood at the time of flare accumulate in the blood following initiation of treatment, suggesting that they are trafficking from inflamed tissues as the inflammation resolves (28). Additionally, the immune complexes and tissue inflammation, together with their associated IFN-production, may be slow to resolve, especially following conventional standard of care treatment, as has been observed in lupus nephritis.

In contrast to the findings observed for T cells and activated innate immune populations, the ISP scores in B cells and LDGs remained stably elevated in flaring SLE patients at all time points following therapy. The reasons for this comparative lack of variation are not clear, but these populations appear to be less susceptible to the effects of potential variations mediated by immune complexes and tissue inflammation.

Given that flow cytometry is available at most academic centers, we investigated whether ISP expression in peripheral blood immune populations had the ability to predict ongoing or recurrent disease activity 1 year later. Although the ISP score and individual ISPs in most populations were associated with disease activity 1 year later, the populations with the strongest predictive ability were those that showed the least variability with disease activity, specifically LDGs, ABCs, and USM B cells. With optimal cutoffs, AUCs for the global ISP score or specific ISPs were in the high acceptable-to-good range and had diagnostic accuracies of 72-76%. We previously found that ABC abundance was associated with longitudinal disease activity with an AUC of 0.66 (28), and that it correlated with global ISG scores (27). Here, we show that measurement of ISP expression in ABCs significantly outperforms measurement of abundance alone and global ISG expression alone in its predictive ability (17).

Recently, CD19 CAR-T cell therapy has shown remarkable efficacy in SLE, resulting not only in prolonged abrogation of disease activity in a significant subset of patients, but also an immunologic reset (7, 42). Studies suggest that a key aspect of this reset is normalization of the IFN signature, which appears to result from significant reductions in the levels of IFN-inducing anti-nuclear antibodies following depletion of autoantibody-producing plasma cells (43, 44). Our data suggests that the efficacy of this therapeutic intervention may be linked, in part, to its ability to normalize ISG expression in B cell populations.

There are several limitations of our study that should be acknowledged. First, although our data support a relationship between IFN signaling and cellular trafficking, the trafficking markers included in our CyTOF panel were not exhaustive due to panel size constraints. Additional integrins and chemokine receptors will be required to more fully characterize tissue-homing potential and distinguish trafficking to specific organ sites. Our cohort also had limited representation of patients with tissue-specific manifestations, including active skin disease, which restricted our ability to directly link trafficking marker expression in circulating cells with organ-specific clinical features. The flaring and quiescent cohorts differed in their racial/ethnic composition, which could contribute to some of the observed differences between groups. However, adjustment for ethnicity did not alter the overall trends observed in the data, with increased ISP expression in flaring patients remaining significantly elevated in most populations. Many of the differences in cell population abundance also remained significant following adjustment for ethnicity. Second, trafficking was inferred from surface marker expression in circulating immune cells, and we did not directly demonstrate movement of ISP^hi^ populations into or out of inflamed tissues. Paired blood and tissue samples from patients could be analyzed using single-cell RNA sequencing, spatial profiling, and paired BCR or TCR sequencing to determine whether circulating ISP^hi^ cells share phenotypic or clonal relationships with tissue-infiltrating populations and to explore the mechanisms that promote ISP expression. This would provide stronger evidence that these cells reflect ongoing tissue inflammation and may help identify circulating markers that better capture tissue-specific flare risk.

## Data Availability

The raw data supporting the conclusions of this article will be made available by the authors, without undue reservation.

## References

[1] Tselios K Gladman DD Touma Z Su J Anderson N Urowitz MB . Disease course patterns in systemic lupus erythematosus. Lupus. (2018) 28:961203318817132. doi: 10.1177/0961203318817132 30526328

[2] Suurmond J Calise J Malkiel S Diamond B . DNA-reactive B cells in lupus. Curr Opin Immunol. (2016) 43:1–7. doi: 10.1016/j.coi.2016.07.002 27504587 PMC5125853

[3] Li Q Wu H Liao W Zhao M Chan V Li L . A comprehensive review of immune-mediated dermatopathology in systemic lupus erythematosus. J Autoimmun. (2018) 93:1–15. doi: 10.1016/j.jaut.2018.07.007 30017673

[4] Mader S Brimberg L Diamond B . The role of brain-reactive autoantibodies in brain pathology and cognitive impairment. Front Immunol. (2017) 8:1101. doi: 10.3389/fimmu.2017.01101 28955334 PMC5601985

[5] Jacobi AM Odendahl M Reiter K Bruns A Burmester GR Radbruch A . Correlation between circulating CD27high plasma cells and disease activity in patients with systemic lupus erythematosus. Arthritis Rheumatism. (2003) 48:1332–42. doi: 10.1002/art.10949 12746906

[6] Tipton CM Fucile CF Darce J Chida A Ichikawa T Gregoretti I . Diversity, cellular origin and autoreactivity of antibody-secreting cell population expansions in acute systemic lupus erythematosus. Nat Immunol. (2015) 16:755–65. doi: 10.1038/ni.3175 26006014 PMC4512288

[7] Mougiakakos D Kronke G Volkl S Kretschmann S Aigner M Kharboutli S . CD19-targeted CAR T cells in refractory systemic lupus erythematosus. N Engl J Med. (2021) 385:567–9. doi: 10.1056/nejmc2107725 34347960

[8] Baechler EC Batliwalla FM Karypis G Gaffney PM Ortmann WA Espe KJ . Interferon-inducible gene expression signature in peripheral blood cells of patients with severe lupus. PNAS. (2003) 100:2610–5. doi: 10.1073/pnas.0337679100 12604793 PMC151388

[9] Bennett L Palucka AK Arce E Cantrell V Borvak J Banchereau J . Interferon and granulopoiesis signatures in systemic lupus erythematosus blood. J Exp Med. (2003) 197:711–23. doi: 10.1084/jem.20021553 12642603 PMC2193846

[10] Bauer JW Baechler EC Petri M Batliwalla FM Crawford D Ortmann WA . Elevated serum levels of interferon-regulated chemokines are biomarkers for active human systemic lupus erythematosus. PloS Med. (2006) 3:e491. doi: 10.1371/journal.pmed.0030491 17177599 PMC1702557

[11] Feng X Wu H Grossman JM Hanvivadhanakul P FitzGerald JD Park GS . Association of increased interferon-inducible gene expression with disease activity and lupus nephritis in patients with systemic lupus erythematosus. Arthritis Rheumatism. (2006) 54:2951–62. doi: 10.1002/art.22044 16947629

[12] Landolt-Marticorena C Bonventi G Lubovich A Ferguson C Unnithan T Su J . Lack of association between the interferon-alpha signature and longitudinal changes in disease activity in systemic lupus erythematosus. Ann Rheumatic Dis. (2009) 68:1440–6. doi: 10.1136/ard.2008.093146 18772188

[13] Niewold TB Swedler WI . Systemic lupus erythematosus arising during interferon-alpha therapy for cryoglobulinemic vasculitis associated with hepatitis C. Clin Rheumatol. (2005) 24:178–81. doi: 10.1007/s10067-004-1024-2 15565395

[14] Kim ST Munoz-Grajales C Dunn SE Schneider R Johnson SR Touma Z . Interferon and interferon-induced cytokines as markers of impending clinical progression in ANA(+) individuals without a systemic autoimmune rheumatic disease diagnosis. Arthritis Res Ther. (2023) 25:21. doi: 10.1186/s13075-023-02997-w 36765391 PMC9912609

[15] Md Yusof MY Psarras A El-Sherbiny YM Hensor EMA Dutton K Ul-Hassan S . Prediction of autoimmune connective tissue disease in an at-risk cohort: prognostic value of a novel two-score system for interferon status. Ann Rheumatic Dis. (2018) 77:1432–9. doi: 10.1136/annrheumdis-2018-213386 29929956 PMC6161671

[16] Mathian A Mouries-Martin S Dorgham K Devilliers H Yssel H Garrido Castillo L . Ultrasensitive serum interferon-alpha quantification during SLE remission identifies patients at risk for relapse. Ann Rheumatic Dis. (2019) 78:1669–76. doi: 10.1136/annrheumdis-2019-215571 31570366

[17] Mai L Asaduzzaman A Noamani B Fortin PR Gladman DD Touma Z . The baseline interferon signature predicts disease severity over the subsequent 5 years in systemic lupus erythematosus. Arthritis Res Ther. (2021) 23:29. doi: 10.1186/s13075-021-02414-0 33451338 PMC7811214

[18] Morand EF Trasieva T Berglind A Illei GG Tummala R . Lupus low disease activity state (LLDAS) attainment discriminates responders in a systemic lupus erythematosus trial: post-hoc analysis of the Phase IIb MUSE trial of anifrolumab. Ann Rheumatic Dis. (2018) 77:706–13. doi: 10.1136/annrheumdis-2017-212504 29420200 PMC5909750

[19] Crow MK . Type I interferon in the pathogenesis of lupus. J Immunol (Baltimore Md 1950). (2014) 192:5459–68. doi: 10.4049/jimmunol.1002795 24907379 PMC4083591

[20] Theofilopoulos AN Baccala R Beutler B Kono DH . Type I interferons (alpha/beta) in immunity and autoimmunity. Annu Rev Immunol. (2005) 23:307–36. doi: 10.1146/annurev.immunol.23.021704.115843 15771573

[21] Hagberg N Ronnblom L . Systemic lupus erythematosus--a disease with a dysregulated type I interferon system. Scand J Immunol. (2015) 82:199–207. doi: 10.1111/sji.12330 26099519

[22] Petri M Fu W Ranger A Allaire N Cullen P Magder LS . Association between changes in gene signatures expression and disease activity among patients with systemic lupus erythematosus. BMC Med Genomics. (2019) 12:4. doi: 10.1186/s12920-018-0468-1 30626389 PMC6327466

[23] Nehar-Belaid D Hong S Marches R Chen G Bolisetty M Baisch J . Mapping systemic lupus erythematosus heterogeneity at the single-cell level. Nat Immunol. (2020) 21:1094–106. doi: 10.1038/s41590-020-0743-0 32747814 PMC7442743

[24] Aringer M Costenbader K Daikh D Brinks R Mosca M Ramsey-Goldman R . 2019 European League Against Rheumatism/American College of Rheumatology classification criteria for systemic lupus erythematosus. Arthritis Rheumatol. (2019) 71:1400–12. doi: 10.1002/art.40930 31385462 PMC6827566

[25] Gladman DD Ibanez D Urowitz MB . Systemic lupus erythematosus disease activity index 2000. J Rheumatol. (2002) 29:288–91. 11838846

[26] Boukhaled GM Gadalla R Elsaesser HJ Abd-Rabbo D Quevedo R Yang SYC . Pre-encoded responsiveness to type I interferon in the peripheral immune system defines outcome of PD1 blockade therapy. Nat Immunol. (2022) 23:1273–83. doi: 10.1038/s41590-022-01262-7 35835962

[27] Faheem Z Boukhaled GM Nassar C Manion K Kim M Bonilla D . Type I interferons enhance B cell activation and promote differentiation of double negative 2 cells in SLE. Lupus Sci Med. (2026) 13:e002042. doi: 10.1136/lupus-2026-002042 42373139 PMC13331238

[28] Manion K Munoz-Grajales C Kim M Atenafu E Faheem Z Gladman DD . Different immunologic profiles are associated with distinct clinical phenotypes in longitudinally observed patients with systemic lupus erythematosus. Arthritis Rheumatol. (2024) 76:726–38. doi: 10.1002/art.42776 38073017

[29] Banchereau R Hong S Cantarel B Baldwin N Baisch J Edens M . Personalized immunomonitoring uncovers molecular networks that stratify lupus patients. Cell. (2016) 165:551–65. doi: 10.1016/j.cell.2016.03.008 27040498 PMC5426482

[30] Weber LM Nowicka M Soneson C Robinson MD . diffcyt: Differential discovery in high-dimensional cytometry via high-resolution clustering. Commun Biol. (2019) 2:183. doi: 10.1038/s42003-019-0415-5 31098416 PMC6517415

[31] Chiche L Jourde-Chiche N Whalen E Presnell S Gersuk V Dang K . Modular transcriptional repertoire analyses of adults with systemic lupus erythematosus reveal distinct type I and type II interferon signatures. Arthritis Rheumatol. (2014) 66:1583–95. doi: 10.1002/art.38628 24644022 PMC4157826

[32] Berlin C Berg EL Briskin MJ Andrew DP Kilshaw PJ Holzmann B . Alpha 4 beta 7 integrin mediates lymphocyte binding to the mucosal vascular addressin MAdCAM-1. Cell. (1993) 74:185–95. doi: 10.1016/0092-8674(93)90305-a 7687523

[33] Kunkel EJ Campbell JJ Haraldsen G Pan J Boisvert J Roberts AI . Lymphocyte CC chemokine receptor 9 and epithelial thymus-expressed chemokine (TECK) expression distinguish the small intestinal immune compartment: Epithelial expression of tissue-specific chemokines as an organizing principle in regional immunity. J Exp Med. (2000) 192:761–8. doi: 10.1084/jem.192.5.761 10974041 PMC2193265

[34] Picker LJ Michie SA Rott LS Butcher EC . A unique phenotype of skin-associated lymphocytes in humans. Preferential expression of the HECA-452 epitope by benign and malignant T cells at cutaneous sites. Am J Pathol. (1990) 136:1053–68. PMC1877421 1693467

[35] Arbones ML Ord DC Ley K Ratech H Maynard-Curry C Otten G . Lymphocyte homing and leukocyte rolling and migration are impaired in L-selectin-deficient mice. Immunity. (1994) 1:247–55. doi: 10.1016/1074-7613(94)90076-0 7534203

[36] de Jesus-Gil C Sans-de SanNicolas L Garcia-Jimenez I Ferran M Celada A Chiriac A . The translational relevance of human circulating memory cutaneous lymphocyte-associated antigen positive T cells in inflammatory skin disorders. Front Immunol. (2021) 12:652613. doi: 10.3389/fimmu.2021.652613 33833765 PMC8021783

[37] El-Sherbiny YM Psarras A Md Yusof MY Hensor EMA Tooze R Doody G . A novel two-score system for interferon status segregates autoimmune diseases and correlates with clinical features. Sci Rep. (2018) 8:5793. doi: 10.1038/s41598-018-24198-1 29643425 PMC5895784

[38] Georgakis S Ioannidou K Mora BB Orfanakis M Brenna C Muller YD . Cellular and molecular determinants mediating the dysregulated germinal center immune dynamics in systemic lupus erythematosus. Front Immunol. (2025) 16:1530327. doi: 10.3389/fimmu.2025.1530327 40070830 PMC11894538

[39] Der E Ranabothu S Suryawanshi H Akat KM Clancy R Morozov P . Single cell RNA sequencing to dissect the molecular heterogeneity in lupus nephritis. JCI Insight. (2017) 2:e93009. doi: 10.1172/jci.insight.93009 28469080 PMC5414553

[40] Kim JW Kwok SK Choe JY Park SH . Recent advances in our understanding of the link between the intestinal microbiota and systemic lupus erythematosus. Int J Mol Sci. (2019) 20:4871. doi: 10.3390/ijms20194871 31575045 PMC6801612

[41] Sarkar MK Hile GA Tsoi LC Xing X Liu J Liang Y . Photosensitivity and type I IFN responses in cutaneous lupus are driven by epidermal-derived interferon kappa. Ann Rheumatic Dis. (2018) 77:1653–64. doi: 10.1136/annrheumdis-2018-213197 30021804 PMC6185784

[42] Muller F Taubmann J Bucci L Wilhelm A Bergmann C Volkl S . CD19 CAR T-cell therapy in autoimmune disease - a case series with follow-up. N Engl J Med. (2024) 390:687–700. doi: 10.1056/NEJMoa2308917 38381673

[43] Garantziotis P Beretta L Lindblom J Moysidou GS Nikolopoulos D Grieshaber-Bouyer R . Differential molecular signatures in response to CD19-CAR T cell therapy compared with conventional pharmacotherapy in systemic lupus erythematosus. Ann Rheumatic Dis. (2026) 85:153–63. doi: 10.1016/j.ard.2025.06.2132 40701862

[44] Wilhelm A Chambers D Muller F Bozec A Grieshaber-Bouyer R Winkler T . Selective CAR T cell-mediated B cell depletion suppresses IFN signature in SLE. JCI Insight. (2024) 9:e179433. doi: 10.1172/jci.insight.179433 38722688 PMC11383166

