# Supplementary material for: Investigating the association between interferons and flares of systemic lupus erythematosus at a single cell level
Source: Front Immunol. 2026 Oct 6;17:1901515. doi: 10.3389/fimmu.2026.1901515 (PMC13680168; doi:10.3389/fimmu.2026.1901515)
Supplement: Supplementary file 1 [file DataSheet1.pdf]

Supplementary Tables and Figures

Supplementary Table 1. Antibodies used for CyTOF analysis

Metal Tag	Marker	Clone	Format	Company
Y(89)	CD45	HI30	Conjugated	Standard Biotools
Cd(111)	CCR6	11A9	Purified	Thermo Fisher
Cd(112)	CD45RO	UCHL1	Purified	Biolegend
Cd(113)	CD10	HI10a	Purified	Biolegend
Cd(114)	CD8	RPA-T8	Purified	Biolegend
Cd(116)	Ki67	Ki-67	Purified	Biolegend
Pr(141)	IL-21R	152512	Purified	RnD Systems
Nd(142)	HLA-DR	L243	Purified	Biolegend
Nd(143)	TLR9	980914	Purified	RnD Systems
Nd(144)	CD38	HIT2	Conjugated	Standard Biotools
Nd(145)	CD4	RPA-T4	Conjugated	Standard Biotools
Nd(146)	IgD	IA6-2	Conjugated	Standard Biotools
Sm(147)	TLR7	533707	Purified	RnD Systems
Nd(148)	CLA	HECA-452	Purified	Biolegend
Sm(149)	FOXP3	236A-E7	Purified	Thermo Fisher
Nd(150)	CD86	IT2.2	Conjugated	Standard Biotools
Eu(151)	CD14	M5E2	Conjugated	Standard Biotools
Sm(152)	CD21	BL13	Conjugated	Standard Biotools
Eu(153)	CD3-EQ	UCHT1	Purified	Biolegend
Sm(154)	IFIT3	1G1	Purified	Origene
Gd(155)	CD27	L128	Conjugated	Standard Biotools
Gd(156)	CXCR3	G025H7	Conjugated	Standard Biotools
Gd(158)	IL-4Ra	25463	Purified	RnD Systems
Tb(159)	CD19	HIB19	Purified	Biolegend
Gd(160)	T-bet	4B10	Conjugated	Standard Biotools
Dy(161)	IRF7	12G9A36	Purified	Biolegend
Dy(162)	β7	FIB504	Purified	Biolegend
Dy(163)	ISG15	851707	Purified	RnD Systems
Dy(164)	CXCR5	RF8B2	Conjugated	Standard Biotools
Ho(165)	PD1-EQ	EH12.2H7	Conjugated	Standard Biotools
Er(166)	Mx1	ERP19967	Purified	Abcam
Er(167)	PKR	6H3A10	Purified	Novus Biologicals
Er(168)	CCR9	L053E8	Conjugated	Standard Biotools
Tm(169)	CD45RA	HI100	Conjugated	Standard Biotools
Er(170)	BST2	RS38E	Purified	Biolegend

Yb(171)	CD123	6H6	Purified	Biolegend
Yb(172)	IgM	MHM-88	Conjugated	Standard Biotools
Yb(173)	CD56	HCD56	Purified	Biolegend
Yb(174)	L-selectin	DREG-56	Purified	Biolegend
Lu(175)	CD11c	Bu15	Purified	Biolegend
Bi(209)	CD16	3G8	Conjugated	Standard Biotools

Supplementary Table 2. Key markers used to identify each cell population

Phenotype	Markers
Naive B cells	CD19+, CD21+, CXCR5+, IgD+, IgM+, CD10-
Trans. B cells	CD19+, IgD^{lo}, IgM^{hi}, CD10+
Act. Naive B cells	CD19+, T-bet+, CD21-, CXCR5+, IgD+, IgM+, CD11c+
ABCs	CD19+, CD27-, IgD-, T-bet+, CD21-, $CD11c^{hi}$
USM B cells	CD19+, CD27+, IgD+, IgM+
SM B cells	CD19+, CD27+, IgD-, IgM-
PBs	$CD19^{lo}$, $CD27^{hi}$, $CD38^{hi}$
Tfh cells	CD3+, CD4+, CD45RO+, CXCR5+, PD1+
Tph cells	CD3+, CD4+, CD45RO+, CXCR5-, PD1+
Mem. CD4+ T cells	CD3+, CD4+, CD27+, CD45RO+
Naive CD4+ T cells	CD3+, CD4+, CD27+, CD45RA+
Mem. CD8+ T cells	CD3+, CD8+, CD27+, $T\text{-}bet^{int}$
Effector CD8+ T cells	CD3+, CD8+, CD45RA+, T-bet+
Naive CD8+ T cells	CD3+, CD8+, CD27+, CD45RA+
Effector DN T cells	CD3+, CD4-, CD8-, CD45RA+, T-bet+
Naive DN T cells	CD3+, CD4-, CD8-, CD27+, CD45RA+
Reg. CD4+/CD8+ T cells	CD3+, CD4+ or CD8+, CD27+, CD45RA+, T-bet+, FOXP3+
Act. CD4+/CD8+ T cells	CD3+, CD4+ or CD8+, CD27+, CD45RA+, CD45RO+
DP T cells	CD3+, CD4+, CD8+, CD27+, CD45RA+, CD45RO+
LDGs	CD16+, CD10+
CD16- NK cells	CD56+, CD16-, CD45RA+, T-bet+, CD38+
CD16+ NK cells	CD56+, CD16+, CD45RA+, T-bet+, CD38+
Act. pDCs	CD123+, CD86+, CD45RO+, CD38+, HLA-DR-
pDCs	CD123+, CD45RA+, CD38+, HLA-DR+
mDCs	CD86+, CD38+, HLA-DR+, CD11c+
Non-class./Int. Mono.	CD14+/-, CD16+, CD45RA+, CD86+, HLA-DR+, CD11c+
Class. Mono. 3	CD14+, $CD86^{hi}$, CD45RO+, CD38+, $HLA\text{-}DR^{hi}$, $CD11c^{hi}$, IgM+
Class. Mono. 2	CD14+, CD86+, CD45RO+, CD38+, HLA-DR+, CD11c+
Class. Mono. 1	CD14+, CD45RO+, CD38+, $HLA\text{-}DR^{lo}$, $CD11c^{int}$

Supplementary Table 3. Demographics of SLE patients and healthy controls at baseline visit

	Flaring patients	Quiescent patients	Healthy controls
n	46	24	18
Age (yrs, median [IQR])	40.9 [34.9-53.6]	45.0 [36.9-60.6]	35.9 [31.8-38.9]
Sex (n female subjects (%))	41 (89%)	18 (75%)	17 (94%)
Clinical SLEDAI-2K (median [IQR])	6.0 [4.0-10.0]	0	
Duration (yrs, median [IQR])	5.4 [1.3-14.3]	6.0 [2.3-25.1]	
**Ethnicity[1] (n (%))**			
Black	18 (39%)	4 (17%)	0
White	9 (20%)	12 (50%)	11 (61%)
Asian	17 (37%)	7 (29%)	6 (33%)
Other	2 (4%)	1 (4%)	1 (6%)
**Serologic Markers (median [IQR])**			
dsDNA	16.0 [3.0-64.0]	6.0 [2.2-21.0]	
C3	0.9 [0.7-1.0]	1.0 [0.9-1.3]	
C4	0.2 [0.1-0.2]	0.2 [0.1-0.3]	
**Treatment**			
Prednisone (mg, median [IQR])	5.0 [0.0-13.8]	0	
Anti-malarial (n (%))	34 (73.9%)	22 (92%)	
**Immunosuppressives (n (%))**			
Any	30 (65%)	11 (46%)	
Mycophenolate	20 (44%)	8 (33%)	
Azathioprine	3 (7%)	3 (13%)	
Methotrexate	3 (7%)	0 (0%)	
Belimumab	2 (4%)	0 (0%)	
Cyclosporine	0 (0%)	0 (0%)	
Tacrolimus	1 (2%)	0 (0%)	
Leflunomide	1 (2%)	0 (0%)	

IQR, intra-quartile range. [1]self-identified based on socially-defined categories of ethnicity.

Supplementary Table 4. Statistical comparison of baseline demographic and treatment characteristics between flaring and quiescent SLE patients

Variable	p value
Ethnicity	0.0449
Sex	0.1686
Prednisone dose	0.1609
Antimalarial use	0.1162
Any immunosuppressive use	0.1336
Mycophenolate use	0.4519
Azathioprine use	0.4056
Methotrexate use	0.5462
Belimumab use	0.5429
Cyclosporine use	1.000
Tacrolimus use	1.000
Leflunomide use	1.000

Note: Categorical variables were compared using Fisher's exact test. Prednisone dose was compared using the Wilcoxon rank-sum test.

Supplementary Table 5. Comparison of differential cell abundance and ISP score analyses with and without adjustment for ethnicity

Phenotype	LogFC (not ethnicity adjusted)	p value (not ethnicity adjusted)	LogFC (ethnicity adjusted)	p value (ethnicity adjusted)
**SLE vs HC: Differential cell abundance**				
ABCs	1.74	0.0001	1.20	0.0036
Act. CD4+/CD8+ T cells	0.32	0.1248	0.41	0.0551
Act. Naive B cells	0.97	0.0112	0.31	0.3912
Act. pDCs	-1.07	0.0007	-0.89	0.0096
CD16- NK cells	-0.48	0.0513	-0.66	0.0097
CD16+ NK cells	-0.77	0.0017	-0.80	0.0019
Class. Mono. 1	1.29	0.0001	1.17	0.0008
Class. Mono. 2	0.32	0.1182	0.28	0.1858
Class. Mono. 3	1.36	<0.0001	1.20	0.0001
DP T cells	-0.05	0.8546	0.18	0.4495
Effector CD8+ T cells	0.57	0.1457	0.47	0.2505
Effector DN T cells	-0.70	0.0138	-0.57	0.0611
LDGs	1.12	0.0242	0.57	0.2607
mDCs	-0.34	0.1425	-0.39	0.1150
Mem. CD4+ T cells	0.25	0.2083	0.19	0.3484
Mem. CD8+ T cells	0.21	0.3807	0.33	0.1790
Naive B cells	-0.33	0.3502	-0.37	0.3134
Naive CD4+ T cells	-0.13	0.5777	-0.04	0.8682
Naive CD8+ T cells	0.12	0.6514	0.18	0.5202
Naive DN T cells	1.02	0.0001	0.75	0.0043
Non-class./Int. Mono.	0.44	0.0889	0.38	0.1667
PBs	0.88	0.0287	0.44	0.2684
pDCs	-0.16	0.4713	-0.21	0.3441
Reg. CD4+/CD8+ T cells	1.67	0.0028	1.38	0.0139
SM B cells	-1.11	<0.0001	-1.04	0.0001
Tfh cells	0.01	0.9675	-0.11	0.7380
Tph cells	0.06	0.8422	-0.14	0.6530
Trans. B cells	2.21	0.0008	1.50	0.0226
USM B cells	-1.06	0.0007	-1.00	0.0026
**Flaring vs quiescent: Differential cell abundance**				
ABCs	1.13	0.0062	0.80	0.0535
Act. CD4+/CD8+ T cells	-0.19	0.3123	-0.10	0.6465
Act. Naive B cells	0.30	0.4328	-0.26	0.4902
Act. pDCs	0.14	0.6530	-0.07	0.8325
CD16- NK cells	-0.50	0.0463	-0.57	0.0346
CD16+ NK cells	-0.29	0.2646	-0.12	0.6644

Class. Mono. 1	1.21	0.0001	1.13	0.0025
Class. Mono. 2	0.39	0.0386	0.39	0.0730
Class. Mono. 3	0.61	0.0351	0.41	0.2035
DP T cells	-0.80	0.0014	-0.33	0.1997
Effector CD8+ T cells	0.29	0.4652	0.76	0.0716
Effector DN T cells	-0.59	0.0434	-0.37	0.2424
LDGs	-0.63	0.1689	-0.74	0.1182
mDCs	-0.46	0.0423	-0.58	0.0197
Mem. CD4+ T cells	-0.07	0.7302	-0.11	0.6161
Mem. CD8+ T cells	-0.47	0.0468	-0.26	0.3180
Naive B cells	-0.68	0.0612	-0.85	0.0205
Naive CD4+ T cells	-0.06	0.7994	-0.01	0.9591
Naive CD8+ T cells	0.19	0.4738	0.26	0.3688
Naive DN T cells	0.17	0.5171	-0.11	0.7072
Non-class./Int. Mono.	-0.14	0.5824	-0.28	0.3209
PBs	0.50	0.2193	0.31	0.4605
pDCs	-0.13	0.5767	-0.03	0.9213
Reg. CD4+/CD8+ T cells	0.55	0.2927	0.15	0.7891
SM B cells	0.20	0.4729	0.13	0.6458
Tfh cells	-0.06	0.8442	-0.14	0.7228
Tph cells	0.33	0.2895	0.39	0.2387
Trans. B cells	-1.47	0.0103	-1.81	0.0017
USM B cells	-0.19	0.5950	-0.44	0.2592
**Flaring vs quiescent: Differential ISP score**				
ABCs	0.13	0.0209	0.12	0.0335
Act. CD4+/CD8+ T cells	0.18	<0.0001	0.13	0.0006
Act. Naive B cells	0.13	0.0059	0.09	0.0452
Act. pDCs	0.18	0.0053	0.14	0.0315
CD16- NK cells	0.23	0.0001	0.15	0.0093
CD16+ NK cells	0.18	0.0004	0.12	0.0093
Class. Mono. 1	0.22	0.0001	0.14	0.0086
Class. Mono. 2	0.16	0.0001	0.13	0.0040
Class. Mono. 3	0.12	0.0043	0.08	0.0685
DP T cells	0.20	<0.0001	0.16	0.0004
Effector CD8+ T cells	0.13	0.0062	0.13	0.0121
Effector DN T cells	0.16	0.0001	0.12	0.0008
LDGs	0.31	0.0008	0.33	0.0007
mDCs	0.26	<0.0001	0.21	0.0004
Mem. CD4+ T cells	0.21	<0.0001	0.15	0.0004
Mem. CD8+ T cells	0.19	<0.0001	0.15	0.0007
Naive B cells	0.13	0.0149	0.08	0.0997
Naive CD4+ T cells	0.22	0.0003	0.18	0.0017

Naive CD8+ T cells	0.25	0.0001	0.20	0.0009
Naive DN T cells	0.25	0.0001	0.20	0.0017
Non-class./Int. Mono.	0.18	<0.0001	0.16	0.0004
PBs	0.20	<0.0001	0.16	0.0004
pDCs	0.25	<0.0001	0.21	0.0002
Reg. CD4+/CD8+ T cells	0.07	0.0011	0.04	0.0470
SM B cells	0.12	0.0053	0.09	0.0335
Tfh cells	0.27	<0.0001	0.18	0.0005
Tph cells	0.19	0.0001	0.16	0.0007
Trans. B cells	0.15	0.0021	0.10	0.0335
USM B cells	0.20	<0.0001	0.12	0.0029

Note: Differential cell abundance was assessed using the diffcyt testDA_edgeR method, which uses edgeR-based generalized linear models of cell population counts. Differential ISP expression was assessed using the diffcyt testDS_limma method, which uses limma-based linear models of population-level marker expression. Models without ethnicity adjustment included disease group as the variable of interest, while ethnicity-adjusted models additionally included ethnicity as a categorical covariate in the design matrix. The corresponding figures showing results without adjustment for ethnicity are presented in Figure 1B for the differential cell abundance analyses (SLE vs healthy controls, flaring vs quiescent SLE), and Figure 2C for the differential ISP score analysis (flaring vs quiescent SLE).

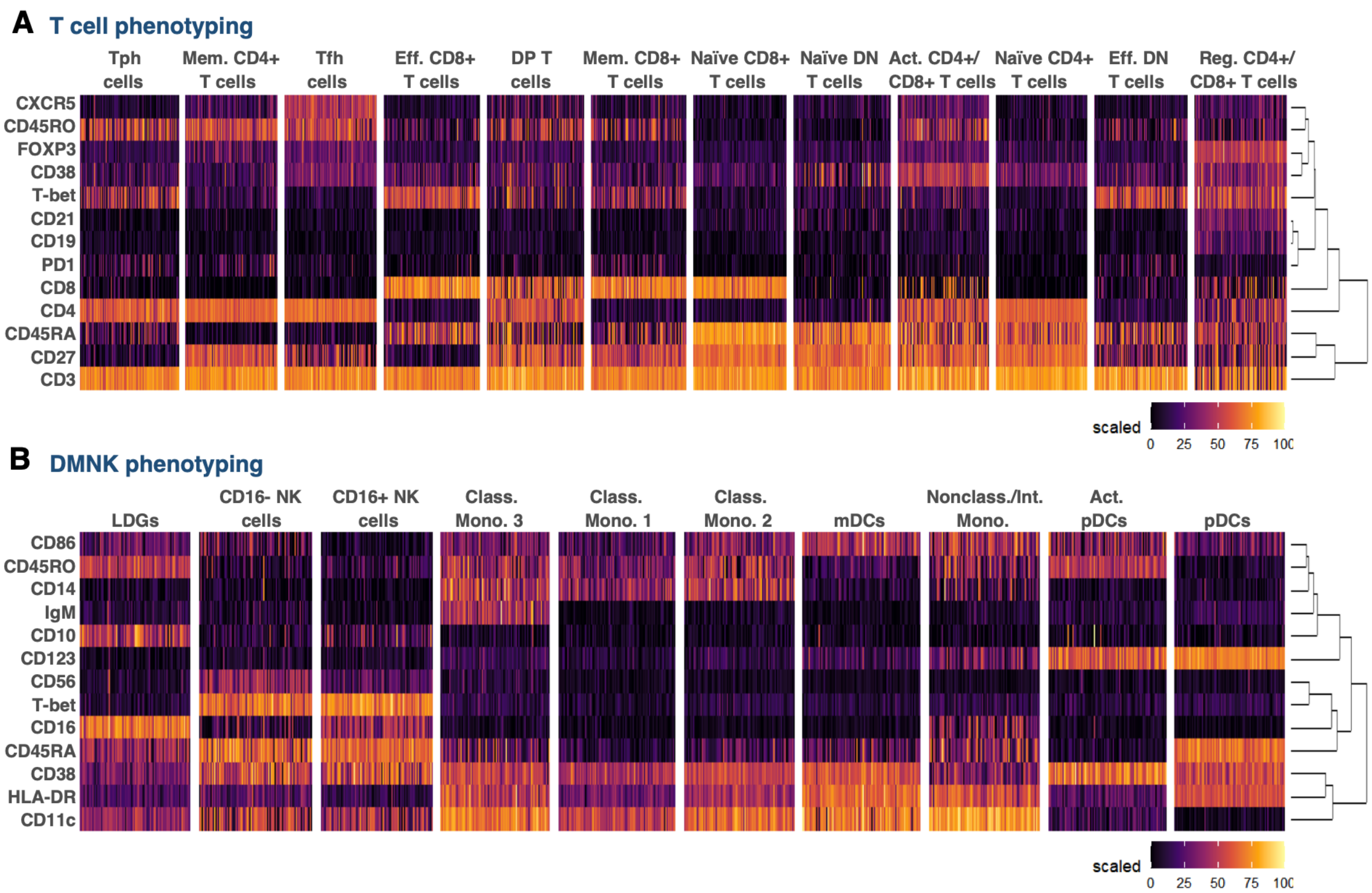

Supplementary Figure 1. Phenotypic characterization of immune cell subsets

A) Heatmap showing expression of phenotyping markers on T cell subsets, scaled relative to other subsets.
B) Heatmap showing expression of phenotyping markers on DC, monocyte, and NK cell subsets, scaled relative to other subsets.

*Heatmap showing expression of phenotyping markers on B cell subsets has been previously published (Faheem at al., *Lupus Science & Medicine*. 2026;13:e002042. https://doi.org/10.1136/lupus-2026-002042).

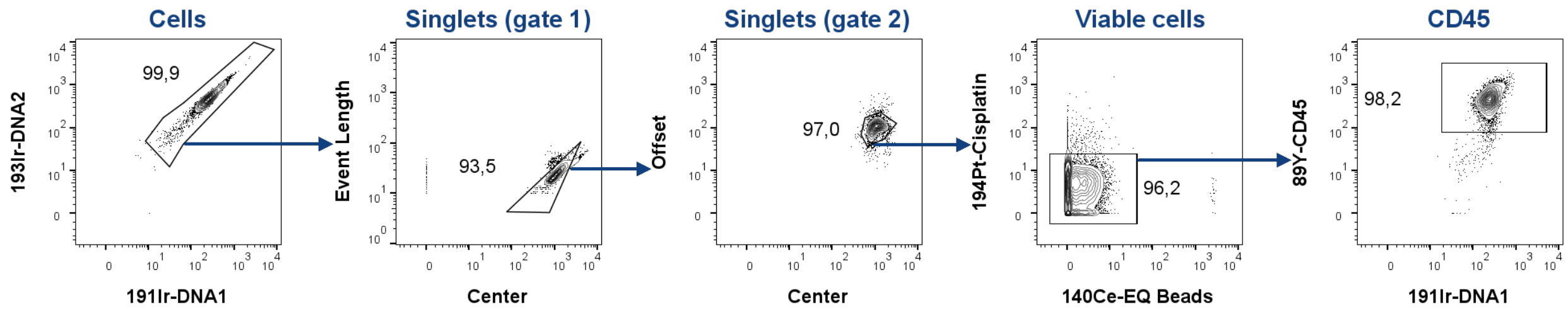

Supplementary Figure 2. Pre-processing gating strategy for CyTOF.
Representative gating hierarchy used to clean raw .fcs files upstream of clustering and analysis. DNA-positive events were identified using the 191Ir-DNA1 versus 193Ir-DNA2 diagonal cloud. Singlets were selected using event length/center and offset/center parameters. Normalization beads and dead cells were excluded, and viable CD45+ cells were selected for downstream graph-based clustering and subsequent phenotyping.

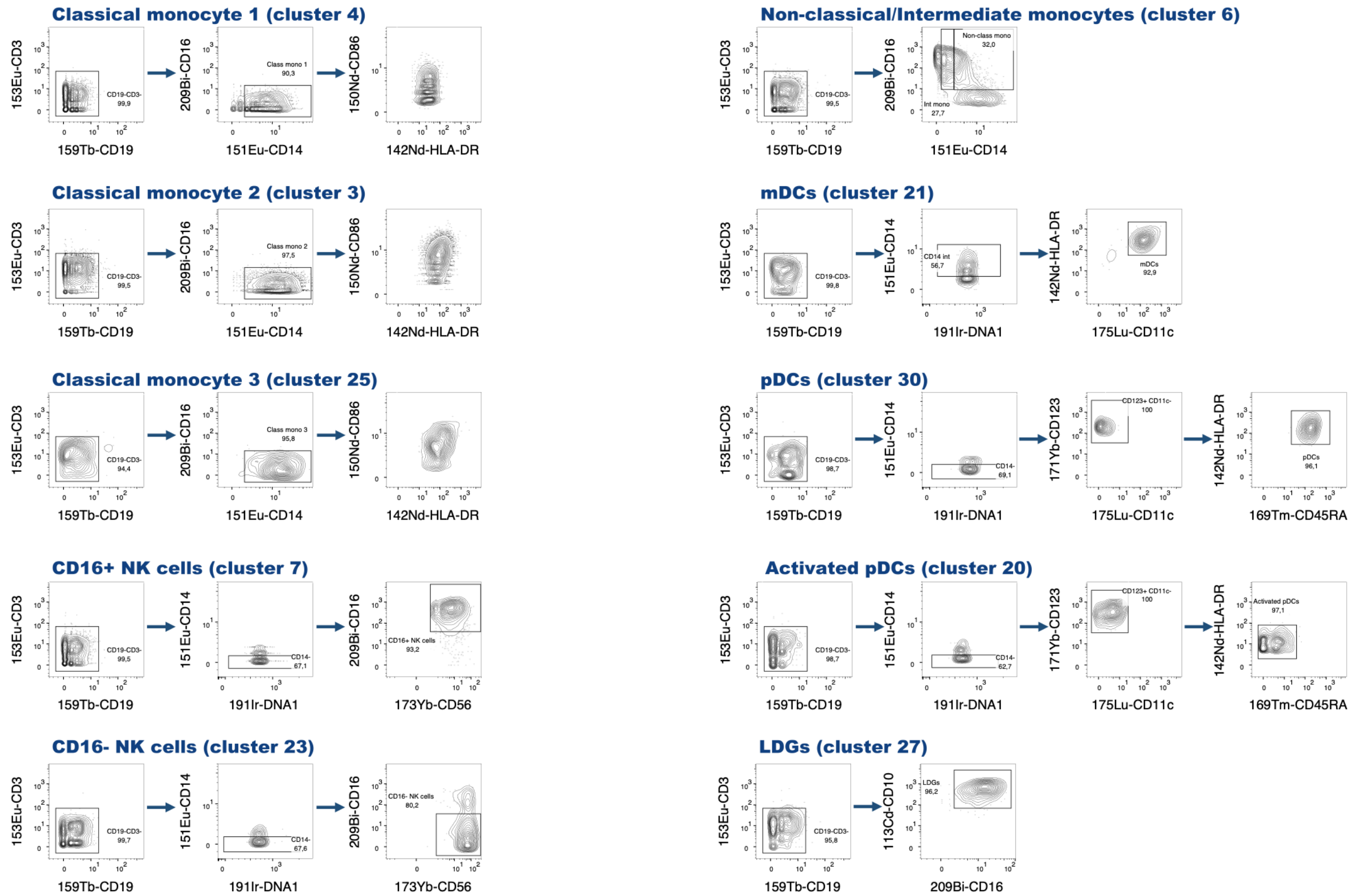

Supplementary Figure 3. Representative gating for monocytes, DCs, NK cells, and LDGs. Following pre-processing to exclude beads, debris, doublets, and dead cells, DNA-positive viable singlets from CyTOF-stained PBMCs were subjected to graph-based clustering using FastPhenograph (KNN = 25). Clustered events were then exported into FlowJo to confirm phenotyping. Representative dot plots show the gating strategy used to identify classical monocyte 1/2/3 populations, non-classical/intermediate monocytes, mDCs, pDCs, activated pDCs, CD16+ NK cells, CD16- NK cells, and LDGs. Classical monocyte 1, 2, and 3 represent Phenograph-derived clusters within the classical monocyte compartment that differ primarily by activation marker expression, including CD86 and HLA-DR, rather than distinct canonical monocyte populations.

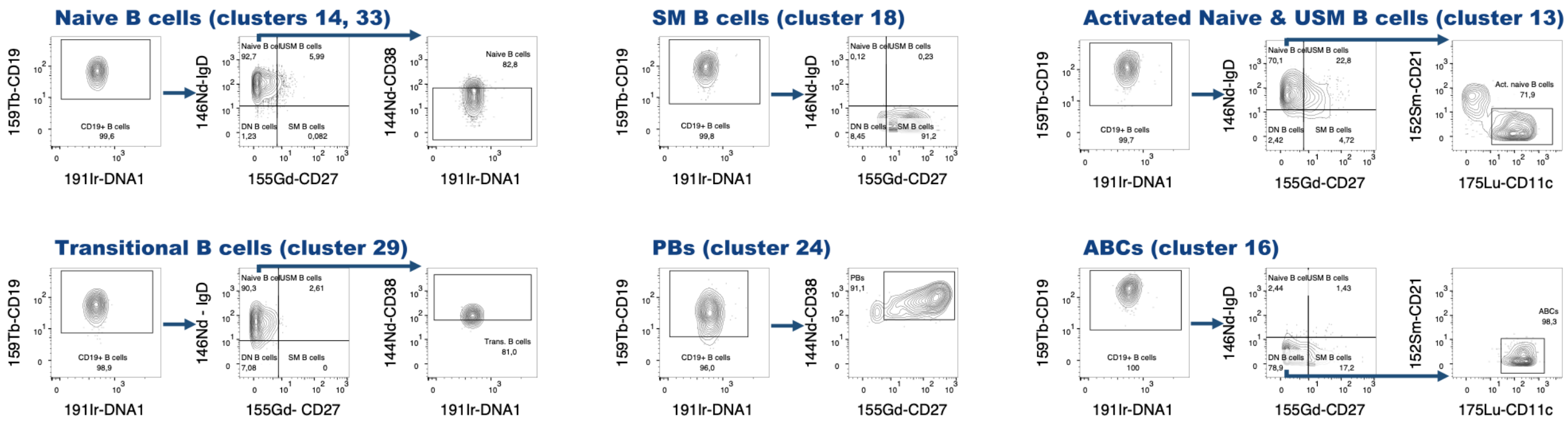

Supplementary Figure 4. Representative gating for B cell subsets.
Representative dot plots show the gating strategy used to confirm phenotyping of B cell clusters exported into Flowjo following graph-based clustering of pre-processed DNA-positive viable singlet lymphocytes. Populations shown include naïve B cells, transitional B cells, activated naïve B cells, unswitched memory B cells, switched memory B cells, age-associated B cells, and plasmablasts.

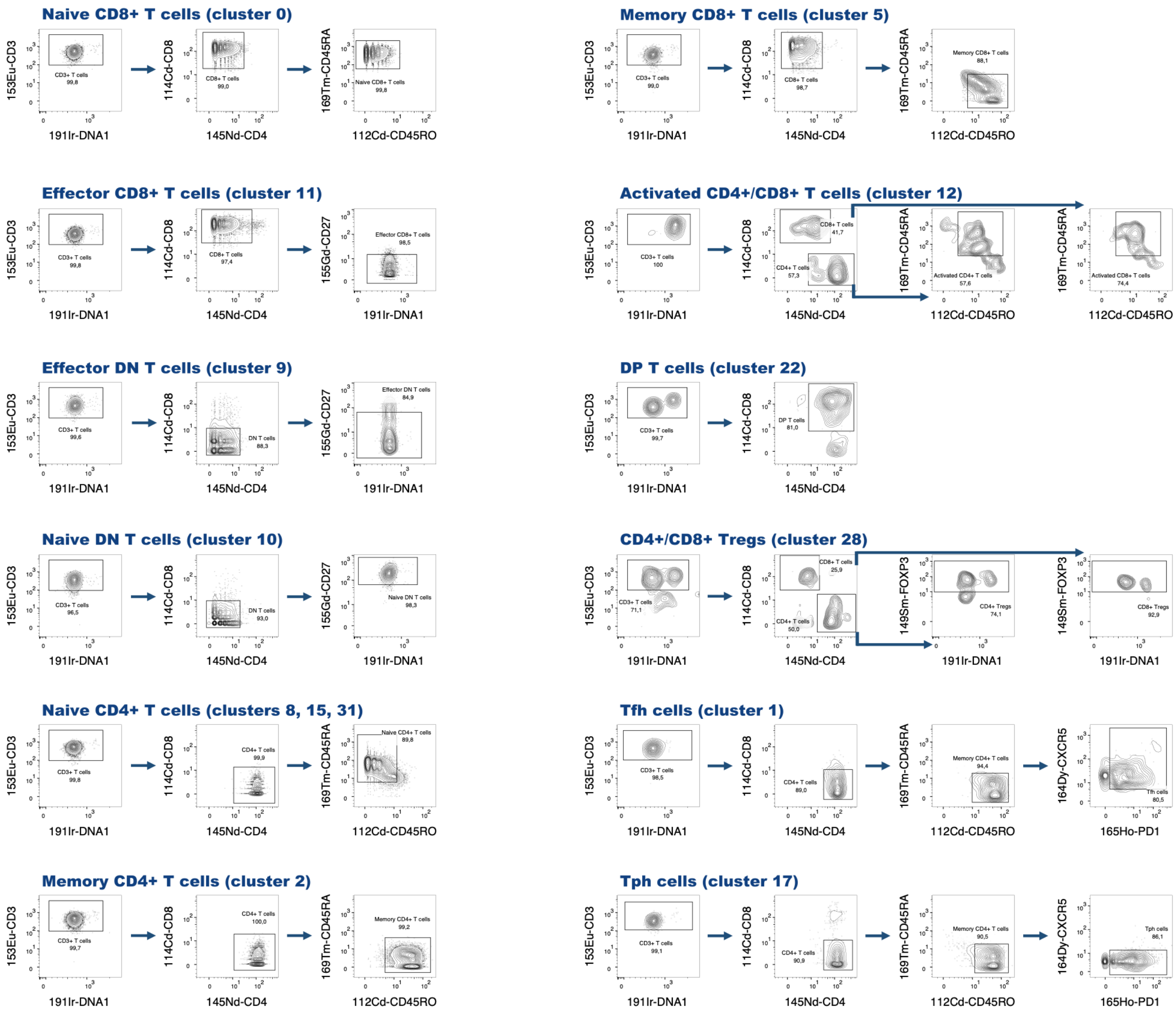

Supplementary Figure 5. Representative gating for T cell subsets.

Representative dot plots show the gating strategy used to confirm phenotyping of T cell clusters exported into Flowjo following graph-based clustering of pre-processed DNA-positive viable singlet lymphocytes. Populations shown include naïve CD8+ and CD4+ T cells, memory CD8+ and CD4+ T cells, activated CD4+/CD8+ T cells, regulatory CD4+/CD8+ T cells, effector CD8+ T cells, naïve and effector double-negative T cells, double-positive T cells, T follicular helper cells, and T peripheral helper cells.

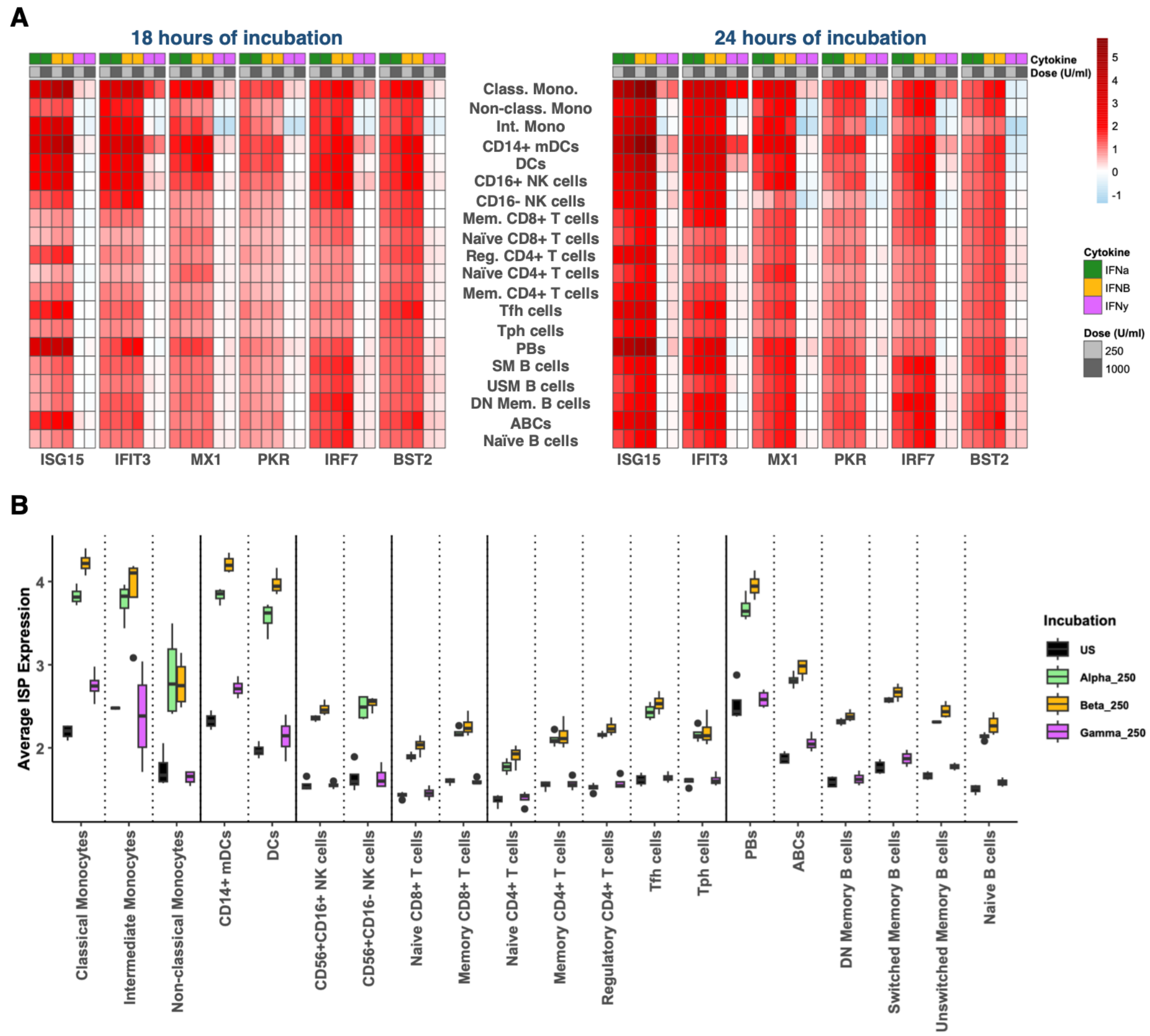

Supplementary Figure 6. Validation of ISPs as surrogate markers of IFN activity.

A) Induction of the six ISPs following incubation of healthy control (HC) PBMCs with the indicated IFNs for 18 or 24 hours. Fold increases shown relative to unstimulated (US) controls. Cells were manually gated on Flowjo as shown in Supplementary Figures 2-5 with some exceptions. Classical monocytes were gated as a single population rather than 3 separate populations because the IFNs induced changes in the levels of molecules used to discriminate between these populations. pDCs and mDCs were analyzed as a single DC population after culture because CD123 and CD11c were upregulated across all DCs, preventing reliable discrimination of these subsets.

B) Mean expression levels of the six ISPs for each cell subset in HC PBMCs incubated with 250 U/mL of the indicated IFNs or no IFN for 18 hours.

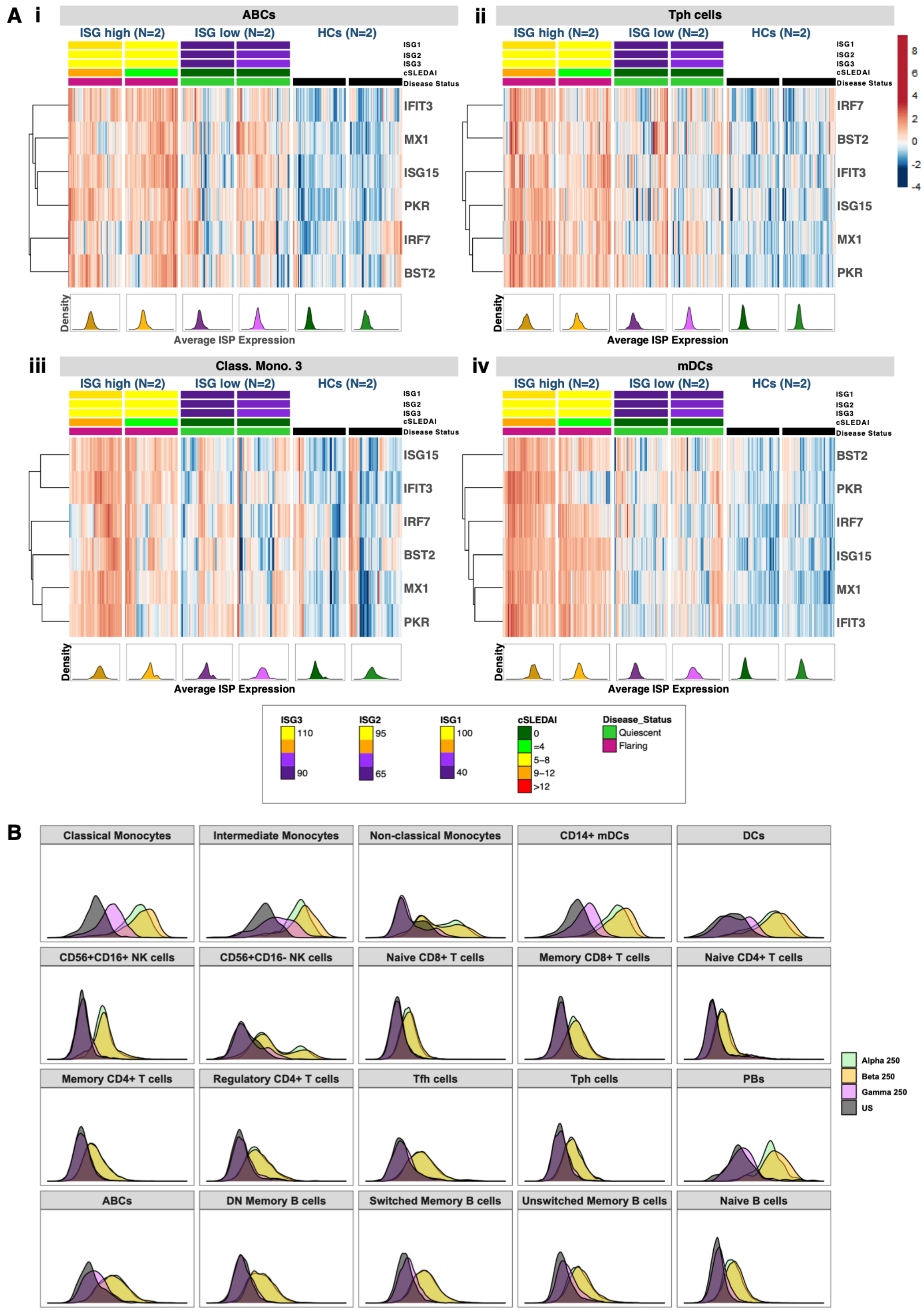

Supplementary Figure 7. Differences in ISP expression patterns in SLE patients and HCs.

A) Heatmaps showing single-cell expression patterns of ISPs in six individual samples with varying degrees of IFN signature across different cell subsets. ISG-high patients were defined as those with ISG scores in the top 15% of all patients, whereas ISG-low patients were defined as those with ISG scores in the bottom 15%. Histograms showing the average expression of the six ISPs for each patient are shown below the heatmaps.

B) Average ISP expression in each cell subset in the PBMCs of a representative HC incubated with 250 U/ml of the indicated IFNs or no IFN (US) for 18 hours.

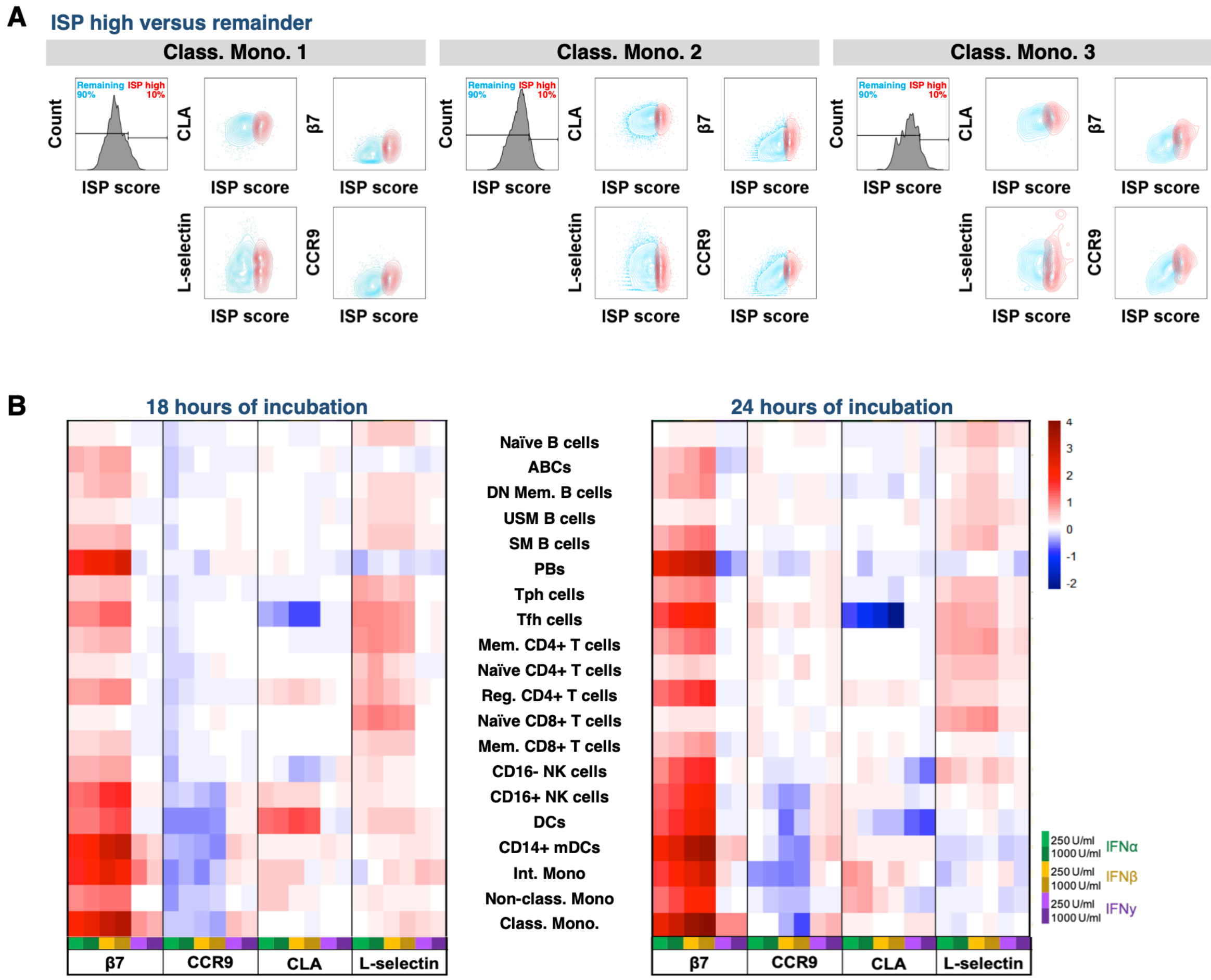

Supplementary Figure 8. Trafficking molecule expression in ISP^{hi} cells and following IFN stimulation

A) Expression of trafficking molecules in the top 10% and remaining 90% of ISP-expressing cells within classical monocyte subsets. ISP^{hi} cells are shown in red, while the remaining cells are shown in blue.

B) Induction of trafficking molecules following incubation of HC PBMCs with the indicated IFNs for 18 or 24 hours. Fold increases are shown relative to unstimulated controls.

A

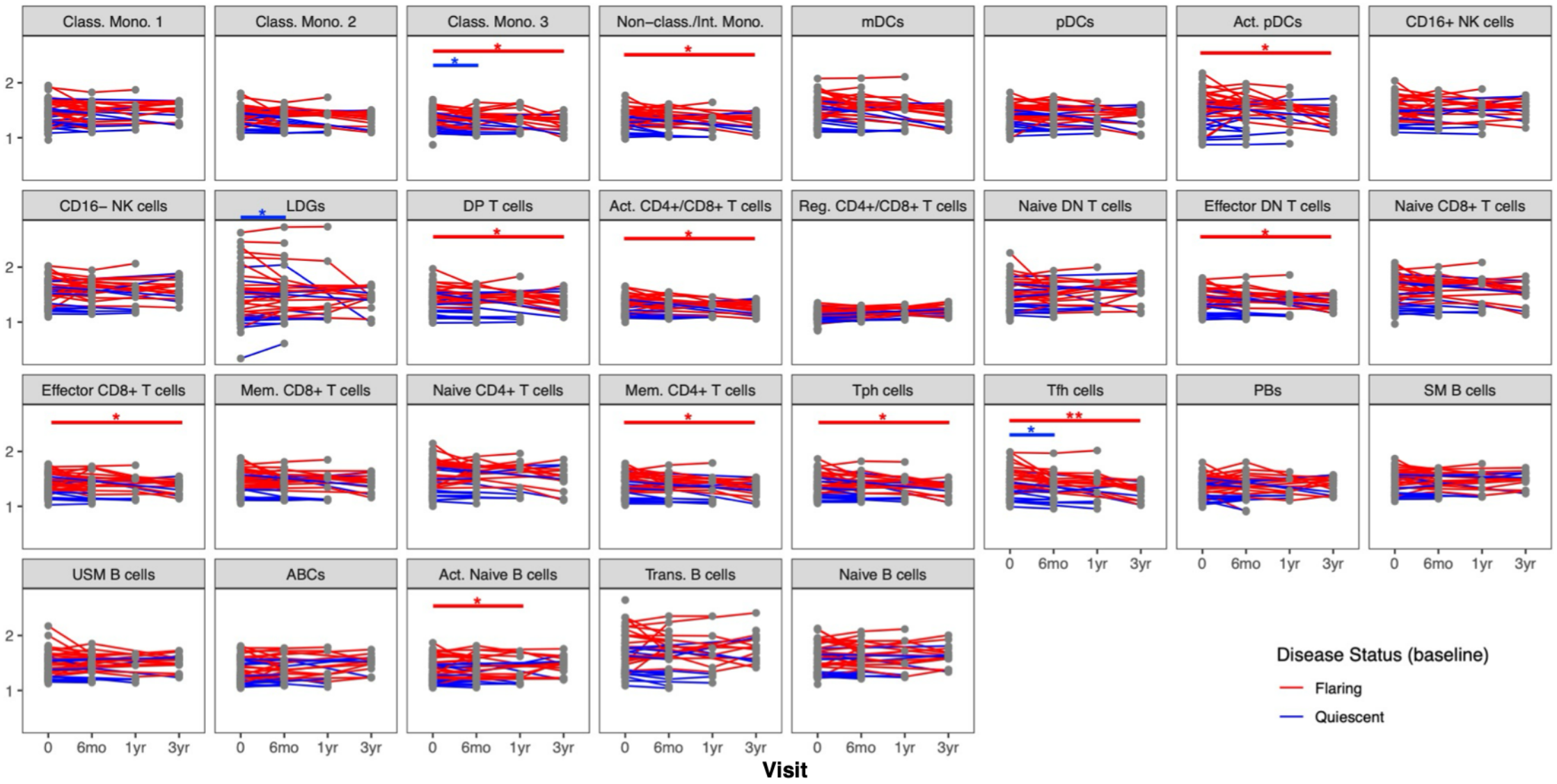

B

Assessment of the prognostic utility of IIPs for predicting subsequent disease flare

Phenotype	Protein Marker	Optimal Cut-off	AUC	Youden's Index	Accuracy	Sensitivity	Specificity	PPV	NPV
LDGs	ISP score	1.506449079	0.762	0.46	0.723076923	0.76	0.7	0.612903226	0.823529412
USM B cells	ISP score	1.496001669	0.77	0.435	0.707692308	0.76	0.675	0.59375	0.818181818
ABCs	ISP score	1.420071681	0.775	0.5	0.738461538	0.8	0.7	0.625	0.848484848
LDGs	PKR	1.160889746	0.8	0.48	0.753846154	0.68	0.8	0.68	0.8
USM B cells	IRF7	1.128365162	0.7835	0.49	0.723076923	0.84	0.65	0.6	0.866666667
ABCs	ISG15	1.205554674	0.7745	0.5	0.738461538	0.8	0.7	0.625	0.848484848

Supplementary Figure 9. Changes in ISP scores over longitudinal follow-up

A) Change in ISP score over time in patients with multiple visits. Patients who were flaring at baseline are shown in red, while patients who were quiescent at baseline are shown in blue. Statistical significance was determined using the Mann Whitney U test, with *$p \le 0.05$, **$p \le 0.01$, ***$p \le 0.001$, and ****$p \le 0.0001$.

B) Table showing the predictive value of ISP levels in LDGs, USM B cells, and ABCs for longitudinal flare.
